# Ecological speciation in European whitefish is driven by a large‐gaped predator

**DOI:** 10.1002/evl3.167

**Published:** 2020-05-13

**Authors:** Gunnar Öhlund, Mats Bodin, Karin A. Nilsson, Sven‐Ola Öhlund, Kenyon B. Mobley, Alan G. Hudson, Mikael Peedu, Åke Brännström, Pia Bartels, Kim Præbel, Catherine L. Hein, Petter Johansson, Göran Englund

**Affiliations:** ^1^ Department of Ecology and Environmental Science Umeå University Umeå SE‐901 87 Sweden; ^2^ Department of Business Administration, Technology, and Social Sciences Luleå University of Technology Luleå SE‐971 87 Sweden; ^3^ Department of Wildlife, Fish, and Environmental Studies SLU Umeå SE‐901 83 Sweden; ^4^ Department of Mathematics and Mathematical Statistics Umeå University Umeå SE‐901 87 Sweden; ^5^ Department of Integrative Biology University of Guelph Guelph ON N1G 2W1 Canada; ^6^ Max Planck Institute for Evolutionary Biology Plön D‐24302 Germany; ^7^ Organismal and Evolutionary Biology Research Program, Faculty of Biological and Environmental Sciences University of Helsinki Helsinki 00014 Finland; ^8^ School of Biological Sciences, Life Sciences Building University of Bristol Bristol BS8 1TQ United Kingdom; ^9^ Evolution and Ecology Program International Institute for Applied Systems Analysis Laxenburg A‐2361 Austria; ^10^ Norwegian College of Fishery Science UiT The Arctic University of Norway Tromsø N‐9037 Norway; ^11^ Climate Impacts Research Centre (CIRC) Abisko Scientific Research Station Abisko SE‐981 07 Sweden

**Keywords:** Body size, ecological speciation, gape size, predator, trade‐off

## Abstract

Lake‐dwelling fish that form species pairs/flocks characterized by body size divergence are important model systems for speciation research. Although several sources of divergent selection have been identified in these systems, their importance for driving the speciation process remains elusive. A major problem is that in retrospect, we cannot distinguish selection pressures that initiated divergence from those acting later in the process. To address this issue, we studied the initial stages of speciation in European whitefish (*Coregonus lavaretus*) using data from 358 populations of varying age (26–10,000 years). We find that whitefish speciation is driven by a large‐growing predator, the northern pike (*Esox lucius*). Pike initiates divergence by causing a largely plastic differentiation into benthic giants and pelagic dwarfs: ecotypes that will subsequently develop partial reproductive isolation and heritable differences in gill raker number. Using an eco‐evolutionary model, we demonstrate how pike's habitat specificity and large gape size are critical for imposing a between‐habitat trade‐off, causing prey to mature in a safer place or at a safer size. Thereby, we propose a novel mechanism for how predators may cause dwarf/giant speciation in lake‐dwelling fish species.

**Impact Summary**

Understanding the mechanisms causing ecological speciation‐with‐gene‐flow is challenging because the phenomenon cannot be directly observed in natural systems. In particular, it has proved difficult to distinguish selection pressures that initiate the speciation process from those appearing at a later stage, as a consequence of the initial niche divergence. We address this problem in three steps. First, we analyze comparative data from 358 populations of European whitefish, and find that ecological speciation in this well‐studied model system is driven by a previously unrecognized selective agent, the northern pike. To understand how this large‐growing predator initiates divergence, we analyze a chronosequence of replicated speciation events (26–10,000 years old). This analysis shows that divergence in a plastic trait, size‐dependent habitat use, leads the way toward reproductive isolation and divergence in genetically controlled traits. Finally, we use an eco‐evolutionary model to analyze the underlying mechanisms. As a result of this combined approach, we propose a previously unrecognized mechanism for how predators can drive dwarf/giant radiations in fish; a remarkably widespread phenomenon that comprises several study systems with a central position in current speciation research. Our results highlight the great utility of combining comparative data with time‐line data to separate causes and consequences of speciation.

For several decades, the question of whether speciation can occur in the face of homogenizing gene flow was hotly debated in evolutionary biology. Today, this debate has shifted focus as research has become more occupied with understanding the processes that cause speciation with gene flow in nature (Nosil 2012; Hendry 2017; Foote 2018). Examples of ongoing ecological speciation in sympatry are especially common in lake‐dwelling fish, as they have an intriguing propensity to form genetically distinct ecotypes that differ in ecology, morphology, and reproductive biology (Skulason and Smith 1995; Seehausen and Wagner 2014). There is substantial variation among ecosystems and species as to how far this divergence has progressed (Hendry et al. 2009; Nosil et al. 2009), but a common feature is the evolution of large‐ and small growing ecotypes along resource and/or habitat gradients in the lake environment. Examples of such ecotypic specialization include threespine sticklebacks (McPhail 1992), African cichlids (Takahashi et al. 2009), rainbow smelt (Taylor and Bentzen 1993), Arctic char (Sandlund et al. 1992), Dolly Varden (Markevich et al. 2018), *Prosopium* sp. (White 1974), and a number of species belonging to the genus *Coregonus* (Svärdson 1979; Mann and McCart 1981; Lu and Bernatchez 1999; Schulz and Freyhof, 2003). Although the processes underlying this pattern have been studied intensively during recent decades (Svärdson 1979; Skulason et al. 1989; Rundle et al. 2000; Knudsen et al. 2006; Vonlanthen et al. 2009; Landry and Bernatchez 2010), a fundamental question remains largely unanswered: why is divergence initiated in some populations and not in others?

To answer this question, we need to improve our understanding of how ecological mechanisms associated with habitat gradients could drive speciation. It is widely accepted that intense intraspecific competition and/or abundant ecological opportunities can cause divergent selection (Bolnick 2004; Landry et al. 2007; Siwertsson et al. 2010; Kahilainen et al. 2011; Wagner et al. 2012; Vonlanthen et al. 2012; Winkelmann et al. 2014; Gordeeva et al. 2015). Other studies suggest that predation (Rundle et al. 2003; Vamosi 2003; Takahashi et al. 2009), spatial variation in temperature (Ohlberger et al. 2008), environmental stress (Symonova et al. 2013), and reduced habitat and prey availability (Landry et al. 2007) can promote divergence. However, the importance of specific selective agents for actually causing speciation still remains elusive. A key problem is that divergence exposes incipient ecotypes to new ecological conditions, and the selective regime can change accordingly over time. For instance, if some ecological mechanism drives individuals to specialize in different habitats, this can cause divergent selection and conspicuous adaptations that are by‐products rather than drivers of the initial divergence.

The best way to avoid confounding the causes and consequences of speciation is to study the process at its earliest stages (Elmer et al. 2010; Seehausen and Wagner 2014; Kautt et al. 2016; Marques et al. 2016; McGee et al. 2016; Marques et al. 2017; Lamichhaney et al. 2018; Marques et al. 2018; Moser et al. 2018). Unfortunately, this approach may lead us to study cases of early population divergence that are unrepresentative of the speciation process, or will never lead to speciation (Nosil et al. 2009, Seehausen and Wagner 2014). This problem, in turn, could potentially be avoided by using comparative analyses (Landry et al. 2007; Siwertsson et al. 2010; Woods et al. 2012; Recknagel et al. 2014) to identify the environmental conditions under which we can expect a future speciation process to proceed. So far, however, these two approaches have rarely been combined.

We addressed these issues by studying whitefish in Scandinavian lakes, where they form genetically distinct ecotype pairs that differ in body size (Svärdson 1979), morphology (Svärdson 1979), resource use (Svärdson 1979; Præbel et al. 2013), and time and place of spawning (Svärdson 1979). Besides from being found in large numbers, these ecotype pairs are typically well known among local fishers (Svärdson 1979), opening up the possibility to use interviews as a method for collecting large amounts of spatial comparative data. Moreover, starting in the late 18th century, there is a richly documented history of anthropogenic introductions that gave rise to new whitefish populations (Burman 1797; Nyström^1863^; Lundberg 1899). Today, the known and variable ages of these young populations provide an excellent opportunity to study how the speciation process initiates and develops over time. In this paper, we present extensive comparative data showing that northern pike is the key driver of ecological speciation in Scandinavian whitefish populations, and use data from populations of different age and modelling to form a hypothesis for why this large‐growing predator is so critically important.

## Methods

### STUDY DESIGN

**Figure 1:**
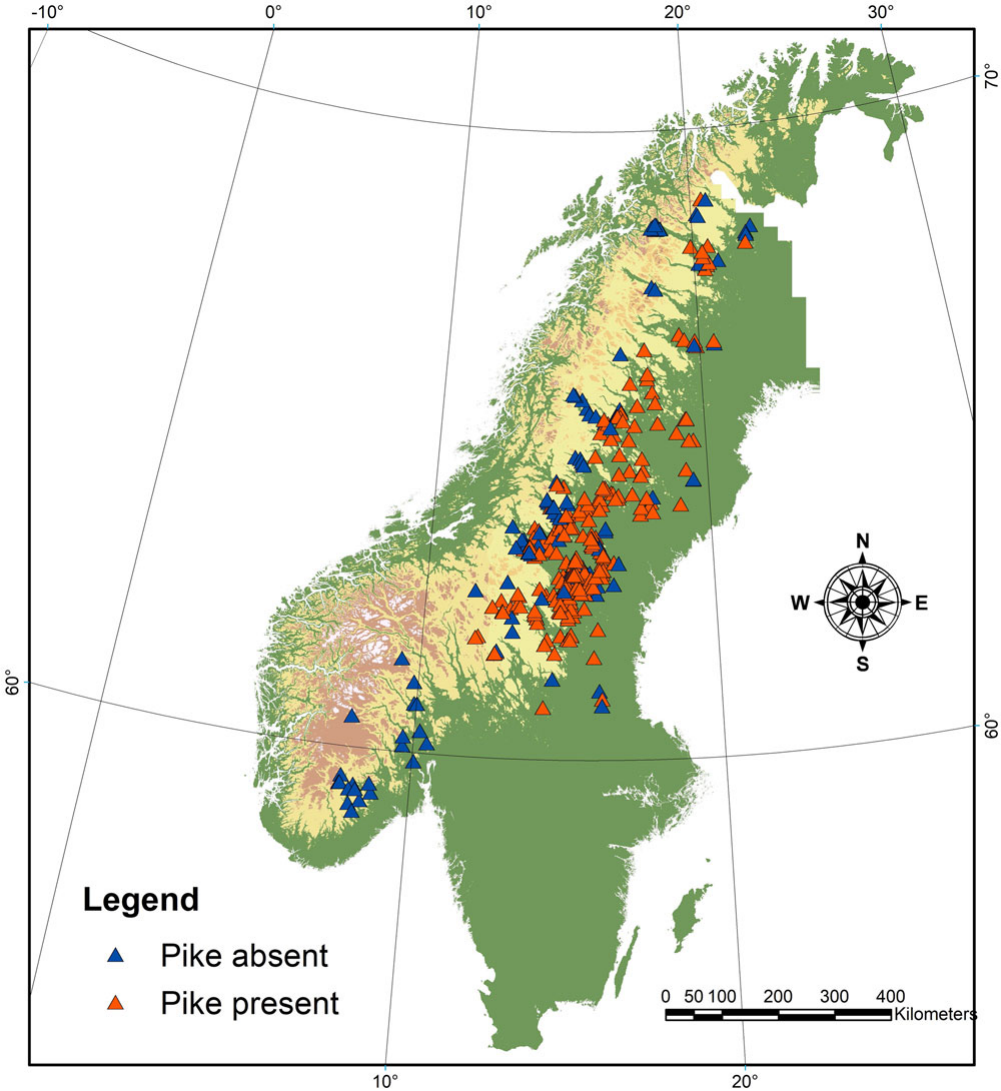
Map of Scandinavia showing the geographical distribution of the 358 lakes in our dataset.

We used data from 358 Scandinavian lakes distributed along a south‐north gradient from southern Norway (58.99 N, 8.29 E) to northern Sweden (68.17 N, 21.97 E; Fig. 1). Interviews with local fishers were performed for all lakes. All other analyses included subsets of these lakes, and for each analysis, we included all lakes for which relevant data were available.

#### Interviews

Local fishers often have detailed knowledge about the habits and spawning sites of whitefish ecotypes in Scandinavian lakes, and co‐occurring ecotypes typically have distinct local names (Svärdson 1979). This allowed us to use interviews to assess large‐scale patterns of maximum body size and the frequency of polymorphism. We asked local fishers (and other persons with relevant knowledge) if the whitefish in a given lake was indigenous or introduced, if there were one or more ecotypes, and for the maximum weight, spawning site and spawning time of each ecotype. We also asked for the composition of the fish community in each lake. Care was taken to follow the same interview protocol for all lakes. To estimate maximum size, we asked about the largest specimen caught in a given lake during the last 25‐year period. We used maximum weight as a crude life history metric because fishers tend to remember this figure and because it effectively captures the divergence between dwarfs and giants. We defined polymorphism as the existence of two or more coexisting populations with different maximum sizes. When deciding whether whitefish populations were polymorphic, lakes were divided into the following four categories: (1) Fishers report two or more populations with different maximum sizes that use different spawning grounds and/or differ in spawning time. (2) Fishers report two ecotypes that differ in maximum size but could not provide information about spawning. (3) Fishers report indications of polymorphism, such as presence of both large and dwarfed spawners and size‐related differences in parasite load, but feel uncertain if these represent different ecotypes. (4) Fishers report that, to the best of their knowledge, there is only one ecotype of whitefish. In the final dataset, we defined lakes from categories 1 (*n* = 100) and 2 (*n* = 53) as being polymorphic and lakes from category 4 (*n* = 199) as being monomorphic. Lakes in category 3 (*n* = 24) were excluded, with the exception of six lakes; four where we had performed standardized sample fishing and two that could be included in our analysis of phenotype‐spawning habitat correlation (see below). Before interviews, we contacted local fishery management organizations to find individuals with deep knowledge about the fish populations in a given lake. On average, we then interviewed 1.46 people per lake (1.44 in pike lakes and 1.56 in pike‐less lakes). Lakes with conflicting information about whether a population was polymorphic were treated as category three (and excluded), unless one (or more) of the interviewed fishermen knew that dwarfs and giants spawn in different places or at different times. In the latter case (*N* = 3), lakes were included in category one.

A subset of the interview lakes (*n* = 48) were used to analyze the association between spawning habitat and phenotype (average body size and gill raker number, see below for more details). For these lakes, we also asked fishers about the water depth at the spawning sites and the average size of spawning individuals.

#### Publications and official records

Data from publications and official records were mainly used to assess the age and origin of populations, and a large proportion of the records of year of introduction in our dataset originate from Swedish and Norwegian reports that were published between 1797 and 2013 (Burman 1797; Nyström 1863; Lundberg 1899; Huitfeldt‐Kaas 1918; Filipsson 1994). If the time of introduction was given in a time span of up to 20 years, we used the middle year. Published data were also included in analyses of differences in neutral genetic markers and gill raker counts (Tables S1 and S2). For the analyses of phenotype‐spawning habitat correlation, we used published information about spawning depth (Svärdson 1951, 1979; Østbye et al. 2005) (*N* = 11) and average body size (Svärdson 1951; Østbye et al. 2005) (*N* = 7) for populations where interviews did not provide this information. Interview information about the composition of fish communities was validated with data extracted from the database PIKE (https://doi.org/10.15468/tx1kgz). As noted in other studies, we found that interview data on the presence/absence of pike and other large and abundant species were reliable (Rask et al. 2000; Spens et al. 2007), whereas small and rare species sometimes were overlooked.

#### Field sampling

To validate interview data and to catch fish for genetic and phenotypic analyses, we performed standardized gillnet sampling in 51 of the interview lakes using 24 benthic gillnets (30 × 1.5 m; eight of multimesh type, four with panels of 33 mm, and 12 with 45 mm mesh size knot to knot) and eight floating gillnets (two of multimesh type [27 m × 6 m] and six single‐meshed nets [30 m × 5 m] with mesh sizes 12, 15, 20, 23, 30, and 38 mm). In a subset of the sampled lakes (*N* = 13), we included two extra floating gillnets with mesh sizes of 33 and 45 mm to allow a comparison of the average size of sexually mature whitefish in the benthic and pelagic habitats, respectively. Additional nonstandardized sampling on spawning grounds was performed with gill netting, hand netting, or ice fishing in 22 lakes (or their adjacent streams).

#### Phenotypic data

The number of gill rakers on the first left gill arch was counted under a dissecting microscope. Even the smallest rudimentary rakers were included in the count. We present gill raker data from ecotype pairs in 72 lakes, out of which 50 had putatively native and 22 had introduced whitefish populations. In 35 of these lakes, the gill raker counts were based on our own samples, and in the remaining 37, we used published data (Table S2). In lakes with more than two ecotypes, we compared the gill raker count of the largest and the smallest ecotype. For the analysis relating average phenotype to spawning habitat (see below), we recorded gill raker means for 10 additional lakes where data were available for only one population. Body length and sexual maturity status were recorded in the field.

#### Genetic data

To identify genetic divergence indicative of reproductive isolation and to investigate the structuring of genetic diversity among and within the introduced whitefish populations, we compared neutral microsatellite genotypic data for ecotypes in 32 lakes. We performed population genetic analyses in 30 of these lakes, and extracted data from the published literature for the remaining two (Østbye et al. 2005) (see Table S1). Eighteen of the analyzed lakes have whitefish populations originating from introductions between 1784 and 1985. One lake (Valsjön) has conflicting information about the introduction date, and 13 lakes have purportedly native whitefish. Individual fish were assigned to ecotype either through sampling on ecotype‐specific spawning grounds or through separation of adult fish based on differences in size and morphology. A detailed account of the genetic analyses is given in Supporting Information.

### STATISTICAL ANALYSES

**Figure 2:**
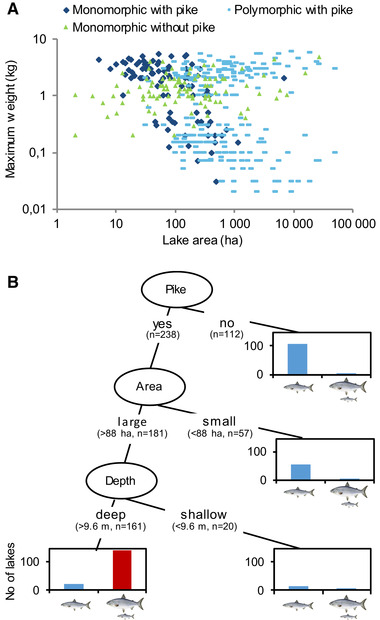
Pike presence, lake area, and maximum depth control the formation of dwarf and giant whitefish ecotypes. (A) Maximum weight (kg) of whitefish from populations in lakes with (*n* = 217) and without (*n* = 103) pike as a function of lake area. Light blue symbols represent polymorphic whitefish populations for which each lake has two corresponding observations. (B) Classification tree (based on 13 explanatory variables, *n* = 350) for the prevalence of polymorphism in whitefish, showing that pike induces co‐occurring dwarf and giant ecotypes in lakes that are large and deep enough. The *y*‐axes show the number of lakes. Cohen's kappa for the whole model was 0.85.

Our interview‐based dataset contains data from 358 lakes, and all noninterview‐based data come from subsets of these lakes. Populations of recent, monomorphic origin cannot be expected to be polymorphic, and may experience rapidly changing growth conditions. Therefore, we did not include whitefish populations introduced after 1960 (the most recent introduction year that has given rise to a polymorphic population according to our interviews) in Figures 2, S1, and S2, and the underlying analyses. For all other analyses, we used the maximum number of lakes that was applicable and for which we had relevant data. This means that the number of lakes included in different analyses vary, either because interviews did not result in complete data for all questions or because noninterview data were not available for all lakes. A detailed description of data selection for specific analyses can be found in Table S3.

For statistical analyses, including linear regression, ANCOVA, and *t*‐test, we scanned residual plots for heteroscedasticity, outliers, and model misspecification. Logarithmic or square root transformations were used to reduce heteroscedasticity and the influence of outlying observations. For logistic regression analyses, we scanned Pearson and deviance residuals for outliers. No outliers or signs of model misspecification were detected. All tests were two sided.

#### Ecological drivers of polymorphism

Relationships between environmental variables and the prevalence of polymorphism were modeled with a classification tree and estimated and cross‐validated with the *rpart* module in R (R Core Team 2018). Thirteen variables were used as predictors: the number of fish species co‐occurring with whitefish, lake area, maximum depth, altitude, temperature sum (total number of degree days above 6°C), and presence/absence of the fish species pike, roach (*Rutilius rutilus*), grayling (*Thymallus thymallus*), burbot (*Lota lota*), Eurasian perch (*Perca fluviatilis*), arctic charr (*Salvelinus alpinus*), brown trout (*Salmo trutta*), and European minnow (*Phoxinus phoxinus*). Optimal tree depth was determined with cross validation and the agreement between data and model predictions was judged with Cohen's κ‐statistics (Cohen 1960). The variable importance of the different predictors included in the analysis is given in Table S4.

#### Divergence versus population age

**Figure 3:**
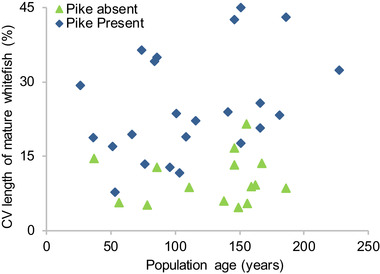
Pike presence drives rapid body size divergence in whitefish. Coefficient of variation for lengths of mature whitefish in lakes with (*n* = 23) and without (*n* = 15) pike as a function of population age.

**Figure 4:**
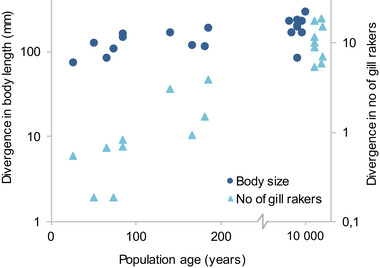
Rapid body size divergence leads the way to gill raker divergence. Between‐cluster differences (based on mature individuals caught in our standardized gillnet surveys, *n* = 19) in average values of body length and gill raker number as a function of population age. The positions of native populations were adjusted along the *x*‐axis to reduce overlap.

**Figure 5:**
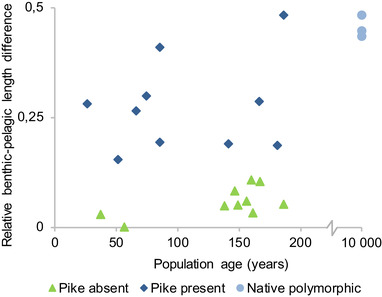
Body size divergence is associated with formation of benthic and pelagic ecotypes. Average length differences of sexually mature whitefish caught in littoral‐benthic and pelagic gillnets as a function of population age. Length differences were calculated as (mean littoral length‐mean pelagic length)/mean littoral length.

In the analysis of CV of individual body lengths (Fig. 3) and the cluster analysis of body length and gill raker data (Fig. 4), we included sexually mature individuals caught in our standardized gillnet sampling. For the between‐habitat comparison of average body length (Fig. 5), we used the combined catch from net types that were represented in both the benthic and pelagic setups (i.e., survey nets and nets with 33‐ and 45‐mm mesh size) and weighted the contribution of each net type to obtain a balanced representation between the two habitats.

For the cluster analyses (Fig. 4), we used the procedure mclustICL in the R module mclust (Scrucca et al. 2016) to identify clusters. Missing data were imputed with the imputeData command in the mix package (Schafer 2017). The differences between mean values for the clusters in a lake were then used as a measure of divergence in body size and number of gill rakers. If more than two clusters were identified, we excluded the intermediate ones. One extremely large individual caught in Stor‐Ringsjön formed its own cluster and was excluded as an outlier.

When gill raker comparisons were made between individuals that were preassigned to ecotype, we compared mature small individuals (<25 cm) and large individuals (>35 cm) caught either on their spawning grounds (dwarf sample from six lakes) or from sampling not associated to spawning grounds (dwarf sample from four lakes and giant sample from all 10 lakes). In one lake (Stor‐Skirsjön), whitefish rarely grow larger than 35 cm, and we therefore compared the mature dwarfs (average length 182 mm) to fish >275 mm.

#### Phenotype‐spawning habitat correlation

Our analysis of the correlation between whitefish phenotype and spawning habitat included populations for which we could get information about spawning habitat and average body size and gill raker number. To ensure that all populations included in the analysis had potential access to all categories of spawning habitat, populations from small and/or shallow lakes (<100 ha, <15 m maximum depth) were excluded. Altogether, 72 whitefish populations from 48 lakes filled these criteria.

Information about spawning depth and habitat was used to categorize populations as stream spawners, shallow lake spawners (depth ≤4 m), or deep lake spawners (depth >4 m). The data were then analyzed with multinomial regression (multinom procedure in the nnet module of R (Venables and Ripley 2002) using average body size and number of gill rakers as predictors and the three spawning categories as response. As the fishers’ estimates of average size could be biased by the type of gear they used, we assessed the robustness of these data by comparing individual interview data points to corresponding average sizes from our own samples. This comparison was partly based on the subset of populations that we had targeted with sampling on their spawning grounds (*N* = 22), using nonstandardized gillnet sampling (*n* = 5), ice fishing (*n* = 3), or hand netting (*n* = 17, i.e., some populations were sampled with more than one method). We also included average sizes from the standardized sample fishing (not performed on spawning grounds) if the given population/ecotype could be separated from the rest of the catch by visual inspection of size and gill raker data (*n* = 21). Regardless of sampling method, the interview data correlated well with our sample data (Fig. S4).

### THE ADAPTIVE DYNAMICS MODEL

We investigated the conditions for divergence in whitefish with an adaptive dynamics approach, using a physiologically structured population model (PSPM, see Metz and Diekmann 1986; de Roos 1997; Claessen et al. 2000; Andersson 2005) in which the population has a continuous size structure and individuals reproduce continuously. Our model contains two habitats—littoral and pelagic—to which whitefish have access at all times. Each habitat has one unique resource type: macroinvertebrates are found in the littoral habitat and zooplankton in the pelagic habitat. An important difference between these resources lies in the way that resource‐use efficiencies for whitefish depend on whitefish size. Although the feeding efficiency for zooplankton has a hump‐shaped relationship to the size of the consumer, it increases almost linearly with whitefish body size for benthic invertebrates (Fig. S5a) (Byström and Andersson 2005). Hence, large whitefish generally depend on benthic invertebrates to sustain positive growth.

However, the benefits of shifting to the benthic resource also depend on size‐ and habitat‐specific mortality rates. Both habitats have equal background mortality rates that are unrelated to size. Because pike is a mainly littoral predator (Chapman and Mackay 1984; Vollestad et al. 1986), pike predation is modeled as an extra mortality rate for individuals feeding on the benthic resource, which decreases with increasing body size (Fig. S5b). Individuals allocate their time in each habitat to minimize the ratio between mortality rate and prey encounter rate (Figs. S5c and S5d). The intake rate of a given foraging strategy is determined by resource type, resource density, and individual size. To keep the model structure conservative and simple, we assume that new recruits are identical. Hence, the only way to become different from other individuals of the same size is by acquired changes in the evolving trait, namely, maturation size. The model is deterministic and does not include a genetic mechanism. Thus, it produces evolutionary divergence under the implicit assumption that assortative mating is present when the population reaches a branching point (or alternatively that reproduction is clonal). A detailed description of the model and parameter values is given in Supporting Information Methods; Figs. S5 and S6; and Tables S5–S7.

## Results

### ECOLOGICAL DRIVERS OF WHITEFISH SPECIATION

Our interviews with local fishers revealed that out of 358 lakes distributed from southern Norway to northern Sweden (Fig. 1), 153 harbored ecotype pairs of dwarf and giant whitefish. These ecotype pairs were generally found in lakes with relatively high species‐richness of fish (Fig. S1), an observation that provides little support for the idea that intraspecific competition and ecological opportunity are the primary drivers of ecological speciation (Stroud and Losos 2016). Instead, our analyses showed that out of 13 analyzed biotic and abiotic variables, presence of northern pike together with lake area and maximum depth determines divergence patterns of whitefish populations in our study area. Pike presence induces divergence into dwarfs and giants in lakes that are large and deep enough (Fig. 2; Table S4; proportion correct predictions = 0.90; Cohen's κ = 0.85). Smaller/shallower lakes have monomorphic whitefish, but the importance of pike for determining whitefish life histories can still be observed. Interview data on maximum weights showed that when pike are present, these lakes have either dwarf or giant whitefish (Figs. 2a and S2).

Whenever fishers had knowledge about the spawning behavior of whitefish in a lake with polymorphism, they reported segregation between dwarfs and giants in time and/or space during spawning. As dwarf and giant whitefish ecotypes typically differ in a range of ecological traits (Svärdson 1979), this suggests that the dwarf/giant pairs reported in our interviews represent cases of incipient ecological speciation. To test this hypothesis and validate the fishers’ observations of polymorphism, we first looked for signs of reproductive isolation between sympatric dwarfs and giants using microsatellite data from 30 lakes where the interviewees had reported dual ecotypes. *F*_ST_‐values were significant between dwarf and giant ecotypes in 23 of these lakes, and the nonsignificant differences between ecotypes were only found in young whitefish populations (introduced between 1825 and 1960, Table S1). Second, we tested for differences in gill raker counts, a trait that is under strong genetic control in European whitefish (heritability *h* ^2^ = 0.79; Svärdson 1979; Bernatchez 2005), and known to be under divergent selection during resource specialization (Præbel et al. 2013; Häkli et al. 2018). When comparing dwarf and giant ecotypes in 70 lakes with reported size polymorphism, we found significant differences in 63 of them (Table S2). Again, nonsignificant differences were only found in young populations. Restricting these analyses to populations that originated before the year 1900, we found that 21 out of 23 ecotype pairs had significant *F*_ST_‐values (mean global *F*_ST_‐value = 0.054; nonsignificant populations were introduced in Bölessjön, 1825 and Sörvikssjön, 1845, Table S1), and that 61 out of 62 ecotype pairs differed significantly in gill raker counts (mean difference = 10.6 rakers, the nonsignificant one was introduced in Bomsjön, 1895, Table S2). Except for in the youngest populations, our data thus show that the dwarf and giant ecotypes that fishers report have developed partial reproductive isolation and substantial differences in an ecologically important, heritable trait.

### THE CHRONOLOGY OF DIVERGENCE

To understand how divergence initiates, we performed standardized gillnet sampling in 38 lakes that have recently introduced whitefish populations (introduced between 1784 and 1985); 23 where pike are present and 15 where they are absent. To only include lakes that are suitable for a future speciation process (see Fig. 2b), these sampling efforts were restricted to lakes that are larger than 100 hectares and deeper than 15 m. First, we scanned the resulting data for signs of initiating body size divergence, and found a strong, rapidly appearing pike effect (Fig. 3). Body size variation among adult whitefish was larger in lakes with pike than in lakes without pike (ANCOVA: pike, *t* = 5.45, *P* < 0.00001, time since introduction, *t* = 1.91, *P* = 0.064, *N* = 38, *r* ^2^ = 0.47; the pike × age interaction was nonsignificant when included, *t* = 1.08, *P* = 0.29).

Next, we used data from the sampled introduction lakes to understand how this rapid body size divergence relates to divergence in other traits. Specifically, we compared the timing of divergence in body size and habitat use, which are highly plastic traits, with that of divergence in gill raker counts and neutral genetic markers. In these analyses, we wanted to exclude any pike‐presence lakes where introductions of multiple genotypes may have contributed to the observed patterns of divergence. We therefore used microsatellite data (available for 18 lakes with known introduction dates) to exclude populations with signs of introductions of multiple genotypes, a procedure that left us with 11 populations with a putatively sympatric signal (Fig. S3).

Going forward with these 11 populations, we first wanted to compare the initial divergence rates of body size and gill raker numbers. In order not to bias the comparison between the two traits, we performed cluster analyses (Scrucca et al. 2016) along the two trait axes simultaneously using the individuals caught in our standardized gillnet sampling. To allow comparison with much older populations, we also included samples from nine lakes with native, polymorphic whitefish.

The analyses gave divergent clusters in all populations except one (Lake Murusjøen, where whitefish were introduced in 1975). Analyzing how between‐cluster differences in body size and gill rakers depend on population age (excluding Murusjøen), we found that divergence in body size is very rapid and precedes divergence in gill rakers (Fig. 4; linear regression, divergence in body size: *t* = 1.84, *N* = 10, *P* = 0.10, *r* ^2^ = 0.30; divergence in number of gill rakers: *t* = 3.29, *N* = 10, *P* = 0.011, *r* ^2^ = 0.57, Slope ± SE = 0.0057 ± 0.0017; both regressions excluding native populations). In fact, a large portion of the body size divergence typically seen in native polymorphic populations is expressed within just a few decades (Fig. 4). Moreover, a comparison between benthic and pelagic catches in the underlying gill net samples showed that this early size divergence is accompanied by an equally rapid divergence in habitat use between dwarfs and giants (Fig. 5; ANCOVA excluding native lakes: pike, *t* = 6.51, *P* < 0.00001, time since introduction, *t* = 1.24, *P* = 0.23, *N* = 20, *r* ^2^ = 0.72). Gill rakers on the other hand show very little divergence between the youngest clusters (Fig. 4), suggesting that differences in body size form the basis for the initial formation of ecotypes.

Next, we tested if gill raker counts differed between ecotypes within the 10 introduction lakes presented in Fig. 4. As the number of gill rakers could not be used for ecotype assignment in these tests, we classified individuals using body size and spawning site. The results from these analyses were consistent with the pattern resulting from our between‐cluster comparisons. Although gill raker numbers did not differ between dwarfs and giants in the youngest populations (introduced after year 1900, *N* = 6, all *t*‐values < 1.68, all *P* > 0.09; Table S2), we found small but significant differences (1.5–2.7 rakers) in four out of four dwarf/giant pairs in the populations that were introduced during the 1800s (*N* = 4, all *t*‐values > 3.22, all *P* < 0.0017; Table S2).

The microsatellite data from these lakes showed a similar pattern: no significant population differentiation between ecotypes in the youngest populations but significant F_ST_‐values between ecotypes in two out of the four older ones (Table S1). Hence, the chronosequence of introduced populations suggests a timeline of divergence where the initial formation of dwarf/giant ecotypes is followed by more slowly appearing differences in gill raker numbers and neutral genetic markers (Figs. 4 and 5; Tables S1 and S2).

Adding the native populations to the chronosequence, the short‐term pike‐driven divergence observed in introduced populations and the long‐term pike‐driven speciation process appear to form a continuum (Figs. 4 and 5). This suggests that we can view divergence in the youngest populations as representing the initial stages of the speciation process. Alternatively, it could be argued that size and habitat divergence may not necessarily lead to heritable differences and reproductive isolation, as has been observed in other fish species that form ecotypes (Gislason et al. 1999; Arbour et al. 2011). However, this does not appear to be the case in our study system. Surveying data from large and deep pike lakes with native whitefish, we did not find a single example of a dwarf/giant pair for which divergence had remained restricted to size (significant gill raker differences in 50/50 lakes, average difference = 11.7; significant *F*_ST_‐values in 13/13 lakes, average *F*_ST_ = 0.061). Hence, even though we lack direct experimental evidence, our data suggest that the initially formed dwarf and giant ecotypes with high predictability will continue to diverge along the speciation continuum.

### THE IMPORTANCE OF BODY SIZE FOR REPRODUCTIVE ISOLATION

**Figure 6:**
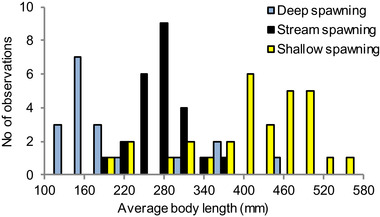
Whitefish spawning behavior is related to body size. Histogram showing the distribution of average body lengths for populations that spawn in stream habitat, shallow lake habitat (depth <4 m), or deep lake habitat (depth >4 m) (*n* = 72).

The hypothesis that size differences lead the way to reproductive isolation implies that the spawning habits of whitefish depend on their body size. To assess the validity of this corollary, we collected information (interview data validated with various kinds of sample fishing, see methods section and Fig. S4 for details) about the average size of sexually mature individuals in all whitefish populations from our study lakes for which data on both spawning habitat and gill raker numbers were available. The resulting data showed that populations of giants typically spawn in shallow lake habitat, whereas more small‐growing populations spawn either in streams or in deeper water in the lakes (Fig. 6). An analysis of this data confirmed that choice of spawning habitat is related to body size but not to gill raker counts (Multinomial logistic regression with stream spawners as reference; body size: stream vs. shallow, *Z* = 3.79, *P* = 0.00015, stream vs. deep, *Z* = 2.13, *P* = 0.033; gill rakers: stream vs. shallow, *Z* = 0.63, *P* = 0.53, stream vs. deep, *Z* = 0.0025, *P* = 1.0, *N* = 72; Fig. 6).

### A MODEL OF PREDATOR‐INDUCED DIVERGENCE IN BODY SIZE AND HABITAT USE

All our empirical results thus point in the same direction: that the strong pike effect on whitefish divergence comes from a unique ability to induce pelagic dwarfs and benthic giants. To understand why pike have this ability as opposed to other potential predators (e.g., brown trout [*Salmo trutta*], arctic char [*Salvelinus alpinus*], and perch [*Perca fluviatilis*]), we must understand (1) how predation and its feedbacks on resource competition among prey can drive divergence into pelagic dwarfs and benthic giants and (2) how this process depends on the characteristics of the focal predator species. Pike are largely restricted to the littoral zone of lakes and stands out by having a gape size large enough to catch relatively large prey (Vollestad et al. 1986; Mittelbach and Persson 1998). To explore the consequences of the presence of a predator with these characteristics, we developed a size‐structured eco‐evolutionary model of the pike‐whitefish system with whitefish maturation size as the evolving trait (see Supporting Information Methods; Figs. S5 and S6; and Tables S5–S7 for a detailed model description). We focused on maturation size because it is an important determinant of growth trajectories (Brett and Groves 1979) that typically differs between sympatric ecotypes in our study system (Svärdson 1979).

**Figure 7:**
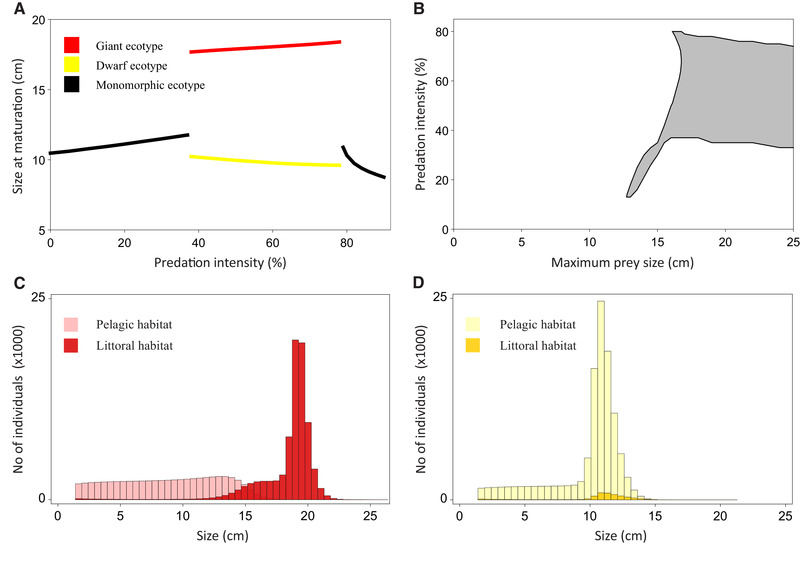
Large‐gaped predators can induce dwarf‐ and giant prey ecotypes by imposing a habitat choice‐growth strategy trade‐off. (A) Model simulation of maturation size as a function of predation intensity from a littoral predator capable of taking prey up to a maximum size of 18 cm. The red line represents giants that mature in the littoral habitat and the yellow line represents dwarfs that mature in the pelagic zone. (B) The range of predation intensities (which can be interpreted as predator density, see Table S6 for details) that induce evolutionary divergence at different values of maximum size of prey that can be taken by the predator. (C) The distribution of the giant ecotype between the pelagic habitat and the littoral habitat at the evolutionary stable state (ESS) when the littoral predator can take prey up to 18 cm and the predation intensity is 70%. The giants mature at 18.2 cm. (D) The corresponding distribution of the dwarf ecotype between the two habitats. Dwarfs mature at 9.7 cm.

The model analyses suggest that habitat‐specific predation can induce evolutionary divergence into dwarfs and giants by imposing a trade‐off that affects life history and habitat choice of prey (Fig. 7a). The presence of pike causes whitefish to either (1) avoid pike in space at the cost of feeding on small pelagic zooplankton that provide limited scope for continued growth (Persson and Brönmark 2002; Byström and Andersson 2005; Shuter et al. 2016) or (2) grow rapidly to reach a size that is subject to low predation risk by delaying the energy‐consuming maturation and using the profitable littoral resource of large benthic invertebrates (Fig. 7c and Fig. 7d). A small‐gaped predator does not impose this kind of trade‐off (Fig. 7b), a result that corresponds well with our empirical data showing no association between whitefish divergence and the presence of small‐gaped predator species such as brown trout, arctic char, and perch. The mechanism behind the strong gape size effect is that when predation risk in the littoral habitat is confined to small prey, pelagic whitefish will be able to reach a size that allows them to shift to the littoral habitat without exposing themselves to high predation risk. Thus, two prerequisites for the necessary life history trade‐off are (1) that the predator is sufficiently large‐gaped to limit the ability of prey to grow out of the predation window when residing in the refuge habitat only and (2) that prey can potentially reach sexual maturity before obtaining a safe size. Hence, besides prey‐resource dynamics, the scope for this kind of predator‐induced divergence will depend on a balance between the gape size of the predator and the inherent growth potential and life history of the prey.

## Discussion

**Figure 8:**
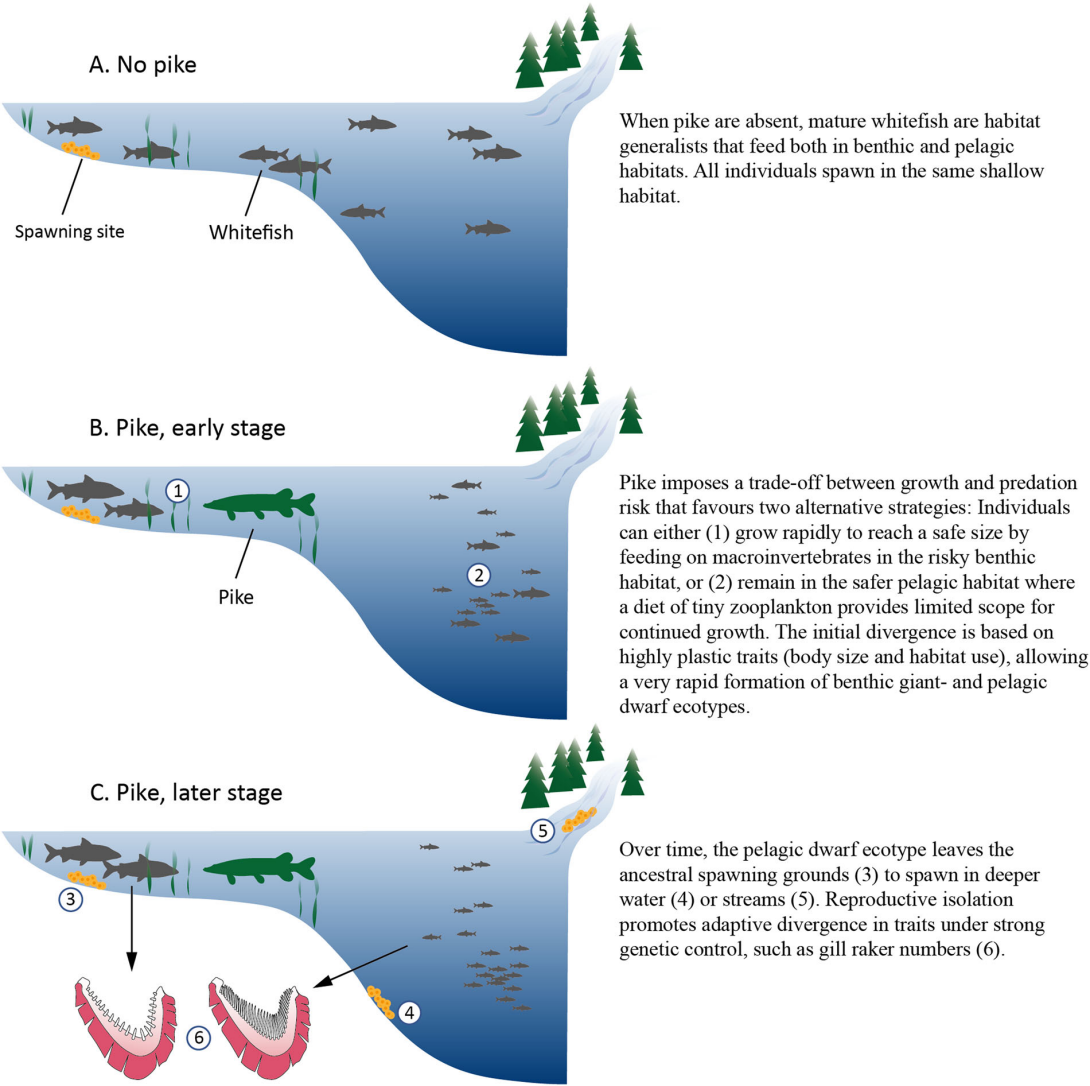
Pike drives ecological speciation in whitefish. An illustration of the mechanisms whereby pike causes phenotypic divergence and ecological speciation in European whitefish.

In this study, we find an answer to the elusive question why benthic‐pelagic ecotype pairs develop in some lakes and not in others. The critical factor that drives ecological speciation along the benthic‐pelagic habitat gradient in this system is a large‐gaped predator, the northern pike. Recognizing pike's role in our study system, we could then target the youngest pike‐exposed whitefish populations to study the initial sequence of trait changes, and use a model rich in the necessary type of ecological detail to analyze the underlying mechanisms. The results suggest that pike drives ecological speciation by inducing pelagic dwarfs and benthic giants; a primary ecotypic differentiation that forms the basis for further divergent adaptations to the respective habitats, and at the same time promotes reproductive isolation (Fig. 8).

Although previous work has shown that both gill raker numbers and body size are under divergent selection during whitefish radiations (Häkli et al. 2018), our data thus suggest that divergent selection on body size and habitat use is the primary route to ecotypic differentiation and subsequent ecological speciation (illustrated in Fig. 8). Although body size divergence has been described as an important component of niche differentiation during ecological speciation in other systems (Schluter 1993; Takahashi et al. 2009), the full, ecological implications of size differences have received relatively little attention in studies of speciation in fish. Unlike other morphological traits, body size determines both an individual's potential gain from feeding on a given food type and its exposure to predation risk while doing so (de Roos and Persson 2013). As a consequence, small and large individuals that face between‐habitat variation in resource gain and predation risk will often specialize on feeding in different habitats (Werner and Hall 1988; L'Abee‐Lund et al. 1993). At the same time, individual growth depends on the density and quality of available resources (de Roos and Persson 2013), and feeding on small or large prey can affect ontogenetic growth trajectories differently (Werner 1988; Shuter et al. 2016). This fundamental property of body size, that is, it both determines and is affected by an individual's ecological niche, is a critical component of the trade‐off that gives body‐size divergence in our model. Hence, our findings are consistent with the idea that phenotypic plasticity is important for speciation (Skúlason et al. 1999; West‐Eberhard 2005; Fitzpatrick 2012; Nonaka et al. 2015).

Through the plasticity of food‐dependent growth, small‐growing individuals can be scared into sacrificing growth opportunities, whereas large‐growing individuals can gain access to resources that allow continued, rapid growth. This way, food‐dependent growth can greatly enhance the adaptive significance of heritable body size variation. In our model, such variation is represented by differences in maturation size: a major source of growth trajectory variation among fish populations (Brett and Groves 1979) and a typical feature of whitefish radiations (Kahilainen et al. 2003). However, any trait variation that affects individual growth could potentially sort individuals along a gradient of size‐dependent resource gain/predation risk. Our model should therefore be viewed as the most straightforward representation of a more general idea: that gape‐limited predation can cause individual prey to either stay in refuge habitat or maximize growth to reach a safe size, depending on their inherent growth potential.

The effect of pike was modified by lake morphology, as body size divergence and subsequent speciation was restricted to pike lakes that are large and deep enough. Specifically, we found that dwarfs do not evolve in small/shallow lakes, suggesting that the pelagic habitat has to be large/deep enough or that the distance to the littoral zone has to be long enough for the pelagic habitat to offer a refuge from pike. Thus, even though the presence of discrete habitats/resources as such do not explain divergence, it seems that a minimum amount of each habitat is required for the pike‐induced process to initiate.

Although our results improve our understanding of how benthic and pelagic ecotypes form, they offer more limited insight into how giants and dwarfs continue to diverge toward speciation: a process that requires assortative mating and some form of heritability that transfers the growth strategies and their spawning behavior between generations. Predation risk could potentially explain the association between body size and choice of spawning sites in much the same way as with size‐dependent habitat choice outside of the spawning season. This remains to be tested, but the association between body size and spawning site nevertheless provides a plausible explanation for why dwarfs and giants develop reproductive isolation over time. Our study thus contributes to a growing body of evidence suggesting that differences in body size may be an important driver of reproductive isolation in polymorphic fish populations (Foote and Larkin 1988; Conte and Schluter 2013). When it comes to the inheritance of adult size, a specific mechanism remains to be demonstrated. It could come from genetically controlled differences in maturation time or size, but there are other possible mechanisms by which size differences could be transferred between generations. For example, size‐dependent choice of spawning sites could feed back on the hatching time and early growth of offspring because the different spawning habitats have different temperature regimes (Lindström 1952; Skulason et al. 1989). Moreover, dwarfs produce smaller eggs than giants (Olofsson 1933), which impedes the initial growth of their offspring (Svärdson and Halvarsson 1968). Demonstrating the mechanisms that cause reproductive isolation between dwarfs and giants will be an important challenge for future research.

The phenomenon that fish populations form sympatric, large‐, and small‐growing ecotypes has been repeated in a large number of species, and along all major habitat axes in lakes (Hindar and Jonsson 1982; McPhail 1992; Sandlund et al. 1992; Kahilainen et al. 2003; Helland et al. 2007; Landry and Bernatchez 2010; Siwertsson et al. 2013). If this parallelism is mirrored in the underlying mechanisms, our results suggest that predation is underestimated as a driver of intraspecific fish diversity in lakes. Although our results apply directly to divergence along the benthic‐pelagic resource axis, the type of trade‐off that gives divergence in our model could appear along any gradient where small prey fish take refuge in suboptimal growth conditions. Such growth conditions can come from spatial variation in a range of environmental variables and do not necessarily depend on the presence of discrete, habitat‐specific resource types. Hence, predator‐induced trade‐offs could potentially explain why dwarf and giant ecotypes also form in situations where diet specialization is less pronounced or even absent (Sandlund et al. 1992; Helland et al. 2008).

To test the hypothesis that predator‐induced growth strategies are an important starting point for ecological speciation, we need to disentangle leaders and followers among the selection pressures and diverging traits that are involved when ecotype pairs form. Our study illustrates how this can be achieved by combining comparative and temporal data, as this can allow us to both identify crucial selection pressures and study their effects on populations over time. Applied to a variety of systems and including a wide range of study methods, this approach holds great promise to improve our understanding of how ecology initiates speciation with gene flow.

## CONFLICT OF INTEREST

The authors declare no conflict of interest.

Associate Editor: Z. Gompert

## Supporting information


**Figure 1**. Dwarf and giant ecotypes of whitefish develop in relatively species rich lakes.
**Figure 2**. Pike presence induces either dwarfs or giants in monomorphic populations.
**Figure 3**. Selection of introduced populations for the chronosequence.
**Figure 4**. Validation of interview data on mean body length.
**Figure 5**. Size‐dependent ecological rates in the different habitats and effects of predation risk on habitat choice in the model.
**Figure 6**. Pairwise invasibility plots for three different predation intensities.
**Table 1**. Genetic data and geographic location for the lakes (*n* = 32) where micro satellites were analyzed.
**Table 2**. Differences between coexisting dwarf and giant whitefish ecotypes in the number of gill rakers (*n* = 72).
**Table 3**. Selection of lakes for the analyses in the paper.
**Table 4**. Variable importance for the predictors included in the classification analyses.
**Table 5**. Description of the model parameters. Superscript numbers within parentheses refers to Supplementary References.
**Table 6**. Description of the model variables and functions.
**Table 7**. Robustness of model results.
