# Supplementary material for: Ecological speciation in European whitefish is driven by a large‐gaped predator
Source: Evol Lett. 2020 May 13;4(3):243–56. doi: 10.1002/evl3.167 (PMC7293097; doi:10.1002/evl3.167)
Supplement: Supplementary file 1 — Figure 1. Dwarf and giant ecotypes of whitefish develop in relatively species rich lakes. Figure 2. Pike presence induces either dwarfs or giants in monomorphic populations. Figure 3. Selection of introduced populations for the chronosequence. Figure 4. Validation of interview data on mean body length. Figure 5. Size‐dependent ecological rates in the different habitats and effects of predation risk on habitat choice in the model. Figure 6. Pairwise invasibility plots for three different predation intensities. Table 1. Genetic data and geographic location for the lakes (n = 32) where micro satellites were analyzed. Table 2. Differences between coexisting dwarf and giant whitefish ecotypes in the number of gill rakers (n = 72). Table 3. Selection of lakes for the analyses in the paper. Table 4. Variable importance for the predictors included in the classification analyses. Table 5. Description of the model parameters. Superscript numbers within parentheses refers to Supplementary References. Table 6. Description of the model variables and functions. Table 7. Robustness of model results. [file EVL3-4-243-s001.docx]

**Ecological speciation in European whitefish is driven by a large-gaped predator**

Gunnar Öhlund, Mats Bodin, Karin A. Nilsson, Sven-Ola Öhlund, Kenyon B. Mobley, Alan G. Hudson, Mikael Peedu, Åke Brännström, Pia Bartels, Kim Præbel, Catherine L. Hein, Petter Johansson, Göran Englund

**Supplementary information**

# **Supplementary Methods**

# **1. An adaptive dynamics model of whitefish divergence**

# 1.1 Model overview

For the theoretical analysis, we use a physiologically structured population model (PSPM, [Metz and Diekmann 1986](#_ENREF_21); [de Roos 1997](#_ENREF_6)), which is based on the models described in Claessen et al. ([2002](#_ENREF_4)) and Andersson ([2005](#_ENREF_1)). We developed the population model to account for predation- and resource-dependent habitat choice and evolution of size at maturation. Individuals are characterized by body size and reside in two separate habitats, corresponding to the pelagic and the littoral zones of a lake. The pelagic habitat has a zooplankton resource, while the littoral habitat has a macroinvertebrate resource and an elevated risk of predation. Individuals balance resource intake rate and mortality risk when choosing the habitat in which to forage. In our model, a niche shift arises as a consequence of adaptive foraging (i.e. individuals shift from feeding on zooplankton to invertebrates as they grow larger). Individuals initially allocate all their energy to maintenance and growth, but after maturation most of the energy is used for maintenance and reproduction. Predation in the littoral habitat is incorporated as a density-independent mortality that decreases with size and approaches zero at the predator’s maximum gape size.

# 1.2 Parameters

Most of the model parameters (Table S5) are estimated for Arctic charr  ([Andersson 2005](#_ENREF_1)) rather than whitefish. We believe that this is justified, as the two species are relatively similar in terms of basic vital rates. One important difference is that whitefish is a more efficient planktivore than charr ([Filipsson and Svärdson 1976](#_ENREF_13)), and continues to feed on zooplankton up to a larger body size than is observed for charr ([Nilsson and Filipsson 1971](#_ENREF_23); [Kahilainen et al. 2003](#_ENREF_18)). Thus, we used 15 g as the optimal body weight for feeding on zooplankton, compared to the value of 7.15 g that has been used for charr ([Byström and Andersson 2005](#_ENREF_3)). The parameter *q*, which represents the survival of the eggs and larvae, has not been measured empirically. We set the survival rate to a relatively low value since egg cannibalism and other types of juvenile mortality are reported to be high in whitefish populations ([Skurdal et al. 1985](#_ENREF_33)). It is noteworthy that changing the survival parameter *q* has the same effects as changing the energy cost of producing one egg (now set to 1). The constants controlling energy allocation between reproduction, growth and metabolism were selected to produce a marked change in energy allocation when individuals mature. The metabolic scaling exponent $\alpha$ was chosen as the median for teleost fish ([Clarke and Johnston 1999](#_ENREF_5)) .

As the model is parameter-rich we did not perform an exhaustive examination of the parameter space. However, as an indication of the robustness of the main result, i.e., that predation results in evolutionary divergence, we provide approximate ranges of parameter values within which this conclusion holds (Table S7). The examination of robustness was limited to parameters that are poorly known.

# 1.3 Ecological dynamics

Brief descriptions of the model parameters, variables, and functions are given in Tables S5-S6 and Fig. S5. The change in the prey density *n(s,t)* of individuals of size *s<s_0_* at time $t$ is given by the McKendrick-von Foerster equation,

$\frac{\partial}{\partial t}n\left( s,t \right)+\frac{\partial}{\partial s}g\left( s,\mathbf{R} \right)n\left( s,t \right)=-d\left( s,\mathbf{R} \right)n(s,t)$ (1)

with the inflow of newborn individuals given by the boundary condition

$g(s_{0},\mathbf{R}\text{)}n\left( s_{0},t \right)= \int_{s_{m}}^{\infty} b\left( s,\mathbf{R} \right)n\left( s,t \right)ds$ (2)

in which *s_0_* is the size at birth and *s_m_* is the maturation size. We denote the zooplankton density in the pelagic habitat by *R_1_*, the macroinvertebrate density in the littoral habitat by *R_2_*, and the resource vector by **R**=(*R_1_*,*R_2_*). The two resources follow semi-chemostat dynamics, i.e., the dynamics of the resource *R_i_* is given by

$\frac{dR_{i}}{dt}=r_{i}\left( K_{i}-R_{i} \right)-V_{i}^{-1}\int_{s_{0}}^{\infty} f_{i}\left( s,\mathbf{R} \right)n\left( s,t \right)ds$ (3)

Numerical simulations of population dynamics were carried out using the Escalator Boxcar Train method ([De Roos et al. 1992](#_ENREF_7); [de Roos 1997](#_ENREF_6); [Brännstrom et al. 2013](#_ENREF_2)).

# 1.4 Adaptive foraging

The ability of fishes to estimate the mortality risk from predators and choose their habitat accordingly has been documented empirically in several species ([Werner et al. 1983](#_ENREF_41); [Tonn et al. 1992](#_ENREF_39); [L'Abee-Lund et al. 1993](#_ENREF_20)). We assume that individuals account for resource availability and mortality risk when selecting a habitat. Specifically, for each size, individuals strive to minimize the ratio of mortality rate to resource encounter rate. We chose this criterion because it is consistent with empirical evidence showing that fishes can balance resource abundance and predation risk ([Gilliam and Fraser 1987](#_ENREF_16)). There is no cost of moving between the two habitats.

We describe adaptive foraging by the fraction of time, *T_i_*(*s*), an individual spends foraging in habitat *i*, which we assume is given by

$T_{i}\left( s,\mathbf{R} \right)\boldsymbol{=}\frac{{{(a}_{i}(s)R_{\boldsymbol{i}}/\mu_{i}(s))}^{\theta}}{{{(a}_{1}(s)R_{1}/\mu_{1}(s))}^{\theta}+{{(a}_{2}(s)R_{2}/\mu_{2}(s))}^{\theta}}$ (4)

The attack rates on the resources and the mortality rate in each habitat are illustrated in Figs. S5a and b. The parameter *θ* describes the strength of habitat selection, where *θ* = 0 indicates random habitat choice, *θ* = 1 means that individuals chose habitat proportionally to the mortality/resource encounter ratio, and *θ >* 1 indicates that individuals favour the habitat with the higher ratio. Figures S5c and d shows the resulting size-dependent habitat use when the habitat selection rule is applied. As the fish grow to a large size, their inability to feed on zooplankton forces a switch to the littoral habitat and a diet of macroinvertebrates.

# 1.5 Energy budget, growth and reproduction

We adopt a net production energy-budget model where metabolic requirements are met first, and the remaining energy is allocated to growth and reproduction. The resource intake rate is given by a Holling Type-II functional response. Assuming the same conversion efficiency, *ε*, in the two habitats, the total energy intake rate from both resources is *εf*(*s*,**R**), cf. Tables S5 and S6. We assume that the metabolic requirements scale allometrically with body weight, *w*. The energy remaining after the metabolic needs have been covered equals

$\varepsilon f\left( s,\mathbf{R} \right)-\beta_{1}W^{\beta_{2}}$ (5)

This energy is divided between growth and reproduction, where the fraction of energy allocated to growth is described by the decreasing function, $K(s)=K_{s_{m}}(s)$, which declines most steeply at the size when maturation is reached. Should the energy required for maintenance exceed the total energy available, we assume that the individual dies from starvation.

# 1.6 Evolutionary dynamics

Adaptive dynamics techniques ([Dieckmann and Law 1996](#_ENREF_8); [Geritz et al. 1998](#_ENREF_15)) were used to investigate the dynamics of the system. Specifically, we assume that the mean population trait value *s_m_* evolves according to a gradient dynamical system known in the adaptive-dynamics literature as the canonical equation ([De Roos et al. 1992](#_ENREF_7)):

$\frac{ds_{m}}{dt}=\frac{1}{2}\rho\sigma^{2}N(s_{m})D(s_{m})$ (6)

Here,*D* is the selection gradient, *ρ* is the mutation probability, *σ*^2^ is the variance of the mutation distribution, and *N*(*s_m_*) is the equilibrium population size. Since *ρ* and *σ*^2^ are positive, the factor *ρσ*^2^/2 only scales the rate of evolutionary change and, therefore, does not affect the location of the evolutionarily stable maturation size. The selection gradient is given by

$D\left( s_{m} \right)=\left. \frac{\partial I_{s_{m}}}{\partial s_{m}^{'}} \right|_{s_{m}^{'}=s_{m}}$ (7)

where $I_{s_{m}}(s_{m}^{'})$ denotes the invasion fitness ([Metz et al. 1992](#_ENREF_22)). The invasion fitness is the long-term exponential per capita growth rate of an initially rare mutant with trait *s’_m_* in a monomorphic population in which the residents mature at size *s_m_*.

At demographic equilibrium, the resource levels, **R**, set by the resident population determine the environment experienced by a mutant. Let Pr(*s*) denote the probability that a newborn mutant maturing at size *s’_m_* survives until it reaches size *s,* in a population consisting of individuals maturing at size *s_m_*, which is given by

$Pr(s)=\exp\left( -\int_{s_{0}}^{s} \frac{d(l,\mathbf{R})}{g_{M}(l,\mathbf{R})}dl \right)$ (8)

where *g_M_*(*l*,**R**) is the ontogenetic growth rate of the mutant and d(*l*,**R**) is the death rate determined by the resident population. We can then calculate the basic reproduction ratio, $R_{0}(s_{m},s_{m}^{'})$, for the mutant as

$R_{0}(s_{m}, s_{m}^{'})=\int_{s_{m}^{'}}^{\infty} \frac{b_{M}\left( s,\mathbf{R} \right)}{g_{M}\left( s,\mathbf{R} \right)}\Pr\left( s \right)ds$ (9)

where *b_M_*(*s*,**R**) is the birth rate of the mutant. Note that *g_M_*(*s*,**R**) and *b_M_*(*s*,**R**), only differ from the resident’s growth and birth rate through a change of maturation size in *𝜅*(*s*), cf. Table S6. The invasion fitness is, in general, given by the dominant Lyapunov exponent of the mutant’s dynamics, but since this is difficult to calculate in practice we use the sign equivalent version, $I_{s_{m}}(s_{m}^{'})=ln\left[ R_{0}\left( s_{m},s_{m}^{'} \right) \right]$. Then the selection gradient equals

$D\left( s_{m} \right)=\left. \left. \frac{\partial ln[R_{0}\left( s_{m},s_{m}^{'} \right)]}{\partial s_{m}^{'}} \right|_{s_{m}^{'}=s_{m}}=\frac{\partial R_{0}(s_{m},s_{m}^{'})}{\partial s_{m}^{'}} \right|_{s_{m}^{'}=s_{m}}$ (10)

since *R_0_*(*s_m_*,*s_m_*) = 1 at demographic equilibrium. A strategy *s_m_* is called an evolutionarily singular if *D*(*s_m_*) = 0. If the invasion fitness has a maximum at a convergence stable singular strategy *s_m_* it is an evolutionarily stable strategy, and if the invasion fitness has a minimum at *s_m_*, it is an evolutionary branching point.

# 1.7 Simulation

We simulated the system as the evolution of size at maturation in a monomorphic population. Before simulation is initiated, there can be one or two convergence stable singular strategies depending on predation intensity (as illustrated by the pairwise invasibility plots in Fig. S6). When there are two convergence stable singular strategies, the larger one is evolutionarily stable, and the smaller one is either a branching point or evolutionarily stable, depending on predation intensity (Figs. S6b and c). The simulation was initiated with a monomorphic population having a maturation size close to the smallest singular strategy, which then was allowed to evolve until it reached a singular strategy. If the singular strategy was a branching point, the monomorphic population was replaced by two populations; one maturing at a slightly larger size and one at a slightly smaller size than the monomorphic population that they replaced. They were then allowed to evolve together until evolutionary stability was reached. Fig. 7a in the main text shows the evolutionary outcome.

**2. Genetic analyses**

Population genetic analyses of sampled whitefish were carried out on genotypes derived from two fully overlapping marker panels comprising nine or 19 polymorphic, di- and tetranucleotide microsatellite loci. Individuals included in the 19 loci data set (36 populations, 16 lakes) formed a fully nested subset within the more extensive nine loci data set (69 populations, 30 lakes). The microsatellite loci used in this study were: *ClaTet1, ClaTet3, ClaTet5, ClaTet6, ClaTet9, ClaTet1, ClaTet12, ClaTet15, ClaTet18,* *Cocl-Lav04, Cocl-Lav06, Cocl-Lav10, Cocl-Lav18, Cocl-Lav27, Cocl-Lav52, Cocl-Lav49, BWF2, ClaTet13, C2-157*. They were amplified in four polymerase chain reaction (PCR) multiplexes in 2.5 μl reaction volume following the PCR protocol and conditions in  ([Præbel et al. 2013](#_ENREF_29)). PCR products were analysed using an ABI 3130XL Genetic Analyzer (Applied Biosystems Inc., Foster City, CA) and fragment lengths were analysed using GENEMAPPER^®^ 4.0 software (Applied Biosystems Inc.).

Deviations from linkage equilibrium (LE) and from Hardy-Weinberg equilibrium (HWE) in the microsatellite data were calculated using the Genepop package in R 3.6.1 ([Rousset 2008](#_ENREF_32)) (10,000 dememorization steps, 100,000,000 Markov chain steps). *P*-values were corrected with the sequential Bonferroni method ([Rice 1989](#_ENREF_31)). To reduce potential biases introduced by the presence of excessively closely related individuals (ECRs), both nine and 19 loci genotype sets were analysed in the R package related ([Pew et al. 2015](#_ENREF_28)). Following simulations, the triadic likelihood estimator  ([Wang 2007](#_ENREF_40)) was used to identify ECR individuals within each population. One of the individuals in each ECR pair was then excluded from all subsequent analyses.

Genetic differentiation between sympatric ecotypes was quantified as pairwise multilocus estimates of *F*_ST_, using Arlequin 3.5.1.2 ([Excoffier et al. 2005](#_ENREF_10)), with 1000 permutations to test significance. To investigate the geographic origins of within-lake genetic diversity in the introduced populations (38 ecotypes, 18 lakes), individual assignment analyses were run using Structure 2.3.4 ([Falush et al. 2003](#_ENREF_11)). Parameters used: 50000 burn-in length, 500,000 MCMC chain replicates, admixture model of ancestry, correlated allele frequencies, population specific alpha prior (starting prior of 0.1). For each K, 10 independent Structure runs were carried out, up to a K of 25. For the Structure results, the true number of distinct genotypic clusters was estimated by selecting the population grouping (K) with the highest log probability of the data (ln Pr(*X*|*K*)). The Structure results were summarized and visualized using CLUMPAK ([Kopelman et al. 2015](#_ENREF_19)) and the R package pophelper 2.2.5 ([Francis 2017](#_ENREF_14)). To corroborate the Structure results, hierarchical relationships among introduced populations were reconstructed using unrooted neighbor-joining (NJ) trees of Cavalli-Sforza cord distances (*D_CH_*), run in Phylip 3.695 ([Felsenstein 1989](#_ENREF_12)). Support for the recovered tree topology was estimated using 1,000 bootstrap replicates. The resulting tree was visualized in FigTree v1.4.2 (http://tree.bio.ed.ac.uk/software/figtree/). For both the Structure and the Phylip analyses, the nine loci genotype set was used for all included populations.

The results of population genetic analyses are summarized in Table S1 (where available, only the results for 19 loci are reported). For the nine loci data set, whilst no loci, overall, where significantly out of HWE, population-specific deviations from HWE were found in 32 out of 621 tests (p<0.05). Pairwise tests of linkage disequilibrium (LD) between loci were found to be significant in 73 out of 2484 tests (p<0.05). For the 19 loci data, no loci were out of HWE overall. For population-specific comparisons significant deviations were found in 34 out of 741 (HWE) and 187 out of 6669 (LD) tests. No obvious over-representation in the identities of specific loci causing deviations in HWE and LD was found among populations and ecomorphs. All significant HWE and LD tests were non-significant following Bonferroni correction.

For the Structure results, the population grouping with the highest log probability was found to be *K* = 16. At this K, patterns of individual cluster assignment within lakes fell into two broad categories (Fig. S3): (i) introduction lakes without a clear signal of secondary introduction (Fig. S3b), and (ii) introduction lakes showing signals of secondary contact between distinct genotypic clusters (Fig. S3c).

NJ tree-based relationships among ecotypes within lakes were consistent with the patterns of individual genetic cluster assignment (Fig. S3a). For the primary divergence lakes, most co-existing species pairs were strongly supported sister species with relatively short branch lengths (bootstrap support 100 %). Exceptions were the clades formed by Oxvattensjön/Rissjön, and Hetögeln/Murusjöen, respectively, which showed strong support (100 %) for monophyly. For Oxvattensjön/Rissjön, this reflects the introduction from the same source population  ([Olofsson 1934](#_ENREF_25)), and for Hetögeln/Murusjöen, it likely reflects that Hetögeln’s dwarf, which spawns in the connecting stream, has spread upstream to Murusjöen. For the lakes included in the secondary contact category, co-existing species generally grouped closest to allopatric populations in other lakes. Only Rosången (50.2 %) and Hökvattnet (77.6 % and 63.1 %) whitefish formed monophyletic groupings, perhaps indicative of more complex secondary contact scenarios with introgression.

The primary divergence lakes were included in the chronosequence of introduced populations used in Figs. 4 and 5. Note that the dwarf/giant ecotype pair in lake Bölessjön was included in the chronosequence even though there is a third, genetically distinct ecotype that was introduced more than hundred years later than the first introduction. The inclusion of this lake was motivated by the apparent lack of introgression between the third ecotype and the original dwarf/giant ecotype pair (Fig. S3b, probably explained by spawning segregation in space (stream vs lake) and in the timing of spawning).

**3. Supplementary discussion**

While the pike effect in our study system is remarkably strong, there are exceptions to the main patterns. In our analysis of the ecological factors that cause whitefish polymorphism, a small number of pike-free lakes (5/153) were classified as polymorphic. A likely explanation for this pattern is recent introductions of multiple genotypes. In fact, additional interviews and archive search showed that introductions of more than one type of whitefish had been performed in at least three of the five lakes. We also cannot exclude the possibility that ecological factors other than pike may be important for driving and/or maintaining polymorphism in these lakes. However, five lakes are by far too few to allow a meaningful analysis aimed at identifying such factors. Furthermore, we found a number of large and deep pike lakes with monomorphic whitefish (23 out of 161 pike lakes in the lower left panel in Fig. 2b). Although there are a range of possible explanations for why speciation may not happen  ([Nosil et al. 2009](#_ENREF_24)), we propose that the limited time for divergence in young populations is a likely explanation. We could establish that 13 of the 23 populations are the result of recent introductions (made between year 1860 and 1975) and it seems likely that this is also true for a fraction of the remaining lakes. It is also likely that sampling error explains some of the observations. Other than that, there may be environmental factors that affect the size/depth threshold where pike will induce polymorphism/speciation. Finally, the results in Fig. 5 could be interpreted as though between-habitat size differences increase over time also in pike-less lakes. We do expect to see a “baseline” difference also in monomorphic populations, as small fish generally are more efficient planktivores, and ontogenetic generalists consequently are more planktivorous during early ontogeny. Hence, the lack of between-habitat size differences in one of the youngest lakes is unexpected. However, we suspect that it is an effect of sampling error. The fact that we found no significant effect of age on divergence in the ANCOVA, and no effect of population age on divergence measured by CV in a larger data set, support this interpretation.

The model predicts divergence into two morphs for intermediate predation intensity and monomorphic populations for either very high or very low predation intensity. Our interpretation is that low predation intensity is insufficient to induce the effect seen at intermediate intensity, whereas very high predation intensities induce too high levels of mortality on the giant morph to allow persistence.

**
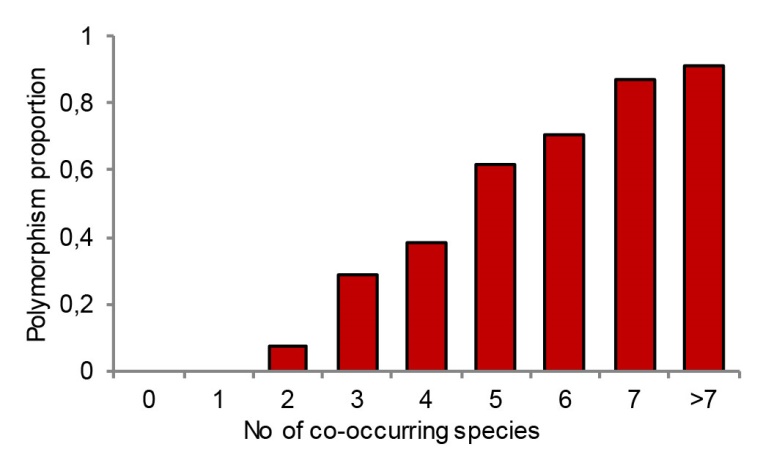
**

**Figure S1. Dwarf and giant ecotypes of whitefish develop in relatively species rich lakes.** The likelihood of finding two or more ecotypes of whitefish increases with fish species richness (logistic regression: coefficient±SE=0.80 ±0.09, Z=8.4, N=350, p<10^-16^). The relationship is also positive if richness is standardized with respect to lake area (coefficient±SE=0.70±0.19, Z=3.77, p=0.0002). Richness was standardized by dividing with Area^c^, where the exponent (c=0.166) was determined by regressing log(species richness) on log(area).


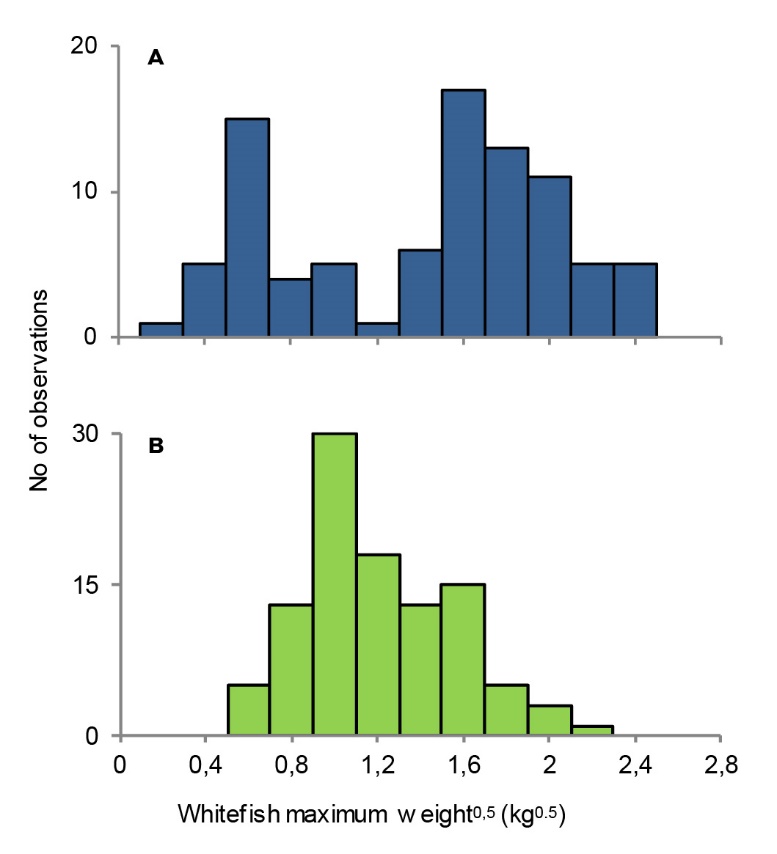


**Figure S2.** **Pike presence induces either dwarfs or giants in monomorphic populations.** Frequency distribution of maximum weights of monomorphic whitefish populations in (**A**) lakes with pike (blue colour, n=88) and (**B**) in lakes without pike (green colour, n=103). Cluster analyses provide support for two clusters in pike lakes (ΔBIC=25.3), and a single cluster in lakes without pike (ΔBIC=4.3).


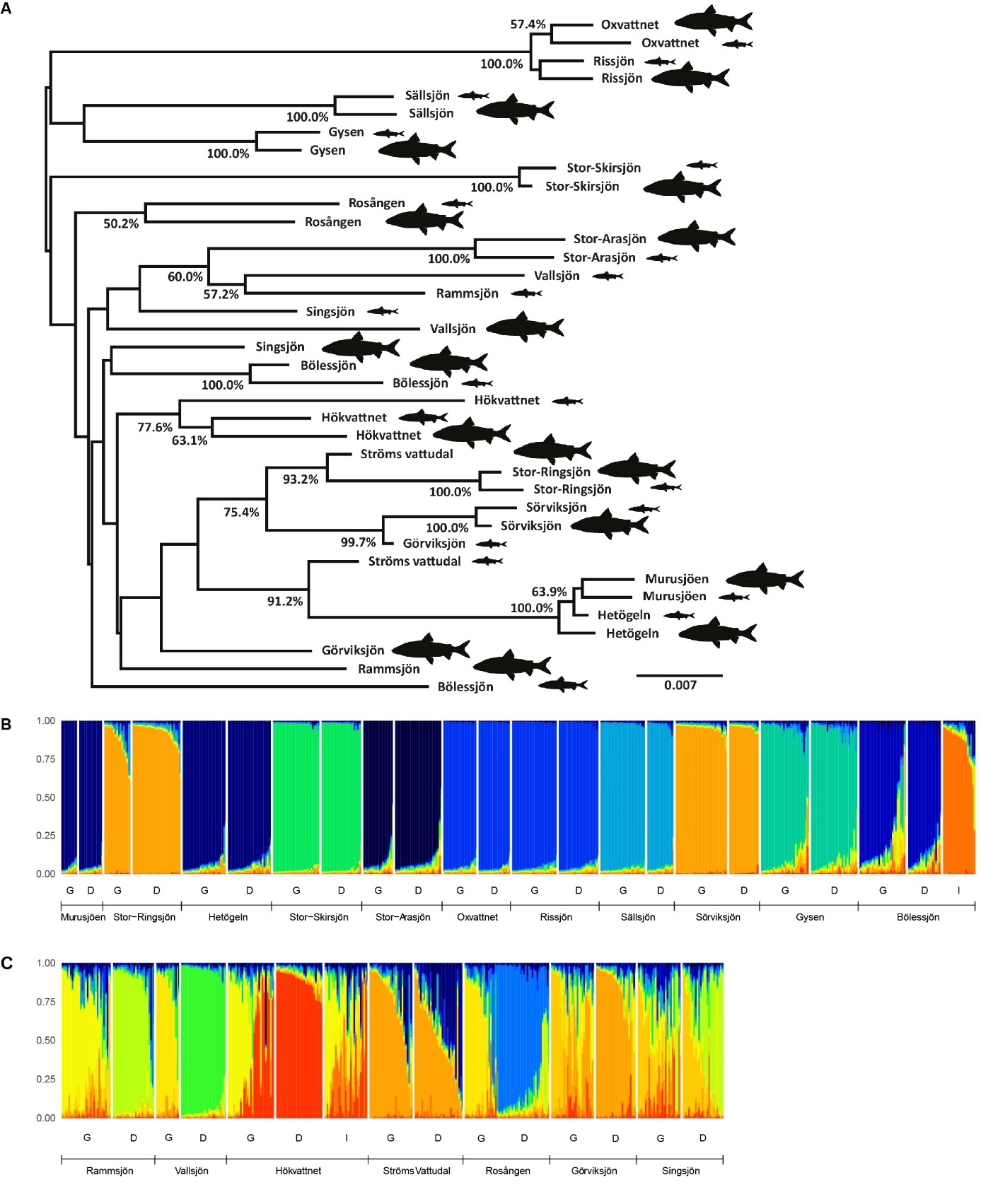


**Figure S3. Selection of introduced populations for the chronosequence.** Genetic data that motivated our decision to include or exclude populations from the chronosequence of lakes with pike and introduced whitefish that were used in Figures 4 and 5 (pike present category). (**A**) Population-based unrooted neighbor-joining tree of Cavalli-Sforza chord distances (*D*_CH_) showing the genetic relationships among whitefish from the different lakes. Numerical values refer to bootstrap support > 50% (1000 iterations). Images of different size at the tips indicate the assigned ecotype: Giant, Dwarf or Intermediate. (**B**) and (**C**) are structure plots showing the populations that were (B) included in, and (C) excluded from the chronosequence. G=giant-, D=dwarf-, and I=intermediately sized ecotypes.


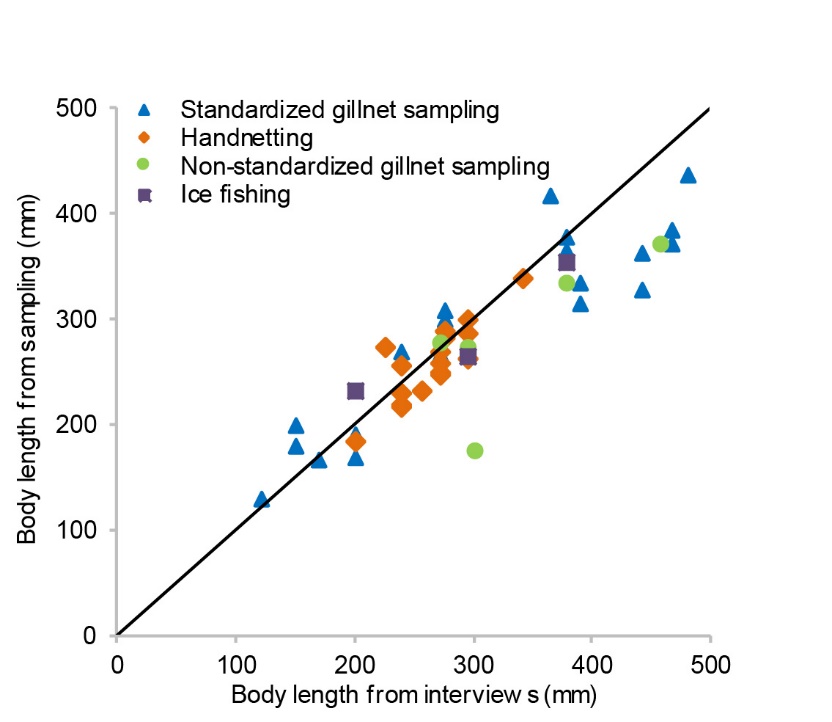


**Figure S4. Validation of interview data on mean body length.** Average body length from our sampling of whitefish populations are compared with corresponding estimates reported by local fishers. The sampling methods include standardized gillnet sampling outside of the spawning season (N=21), as well as hand netting (N=17), gill netting (N=5) and ice fishing (N=3) on spawning grounds.

**
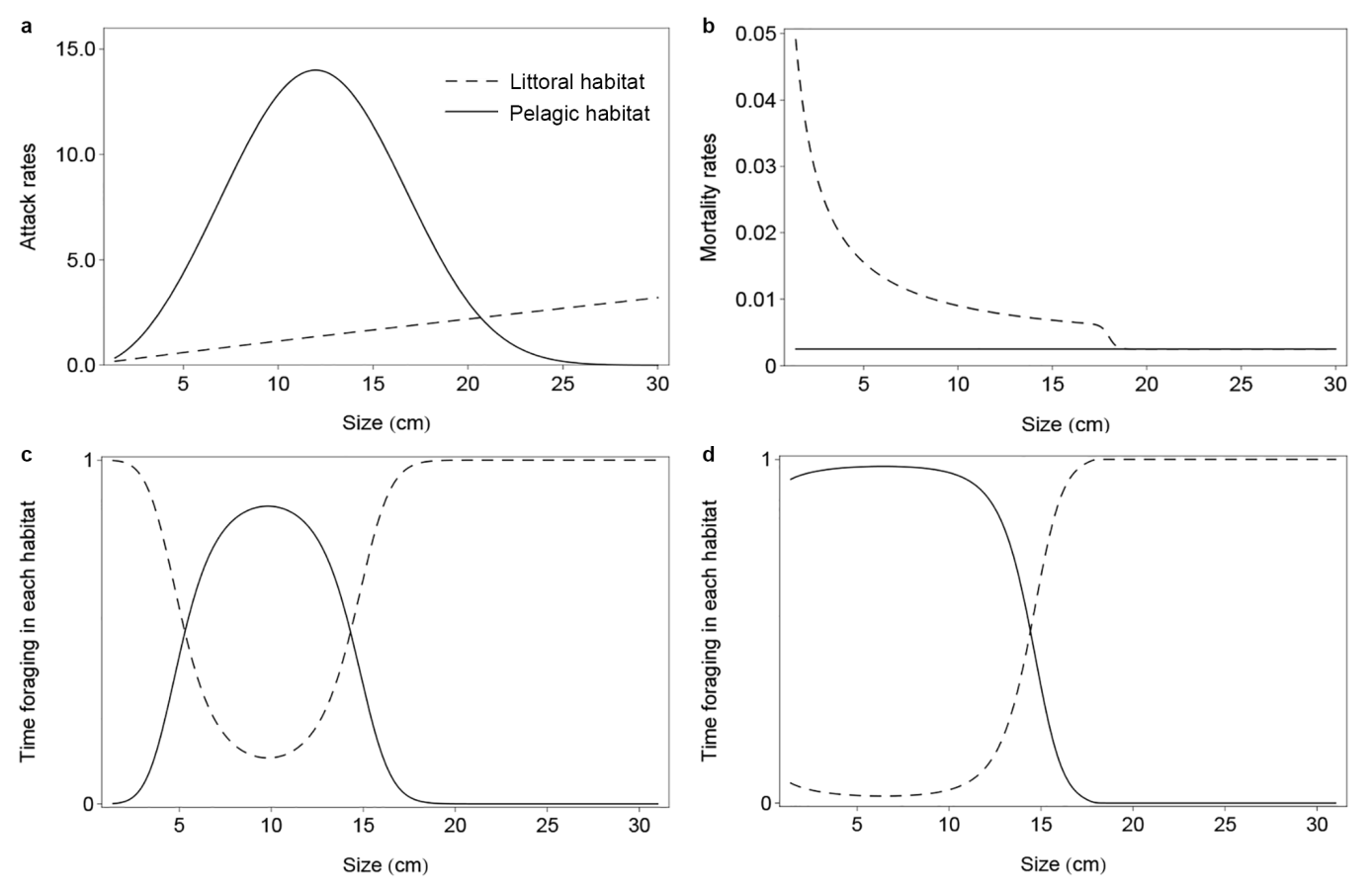
**

**B**

**A**

**C**

**D**

**Figure S5. Size-dependent ecological rates in the different habitats and effects of predation risk on habitat choice in the model.** (**A**) Attack rate on pelagic and littoral resources. (**B**) Mortality rates in the pelagic and littoral habitats. (**C**) and (**D**) Fraction of time spent foraging in the pelagic and littoral habitats without predation (C) and with predation (D). In this simulation, maturation size is 14 cm, maximum prey size eaten by the predator is 18 cm, and the predation intensity is 80%. The functions are specified in Table S6.


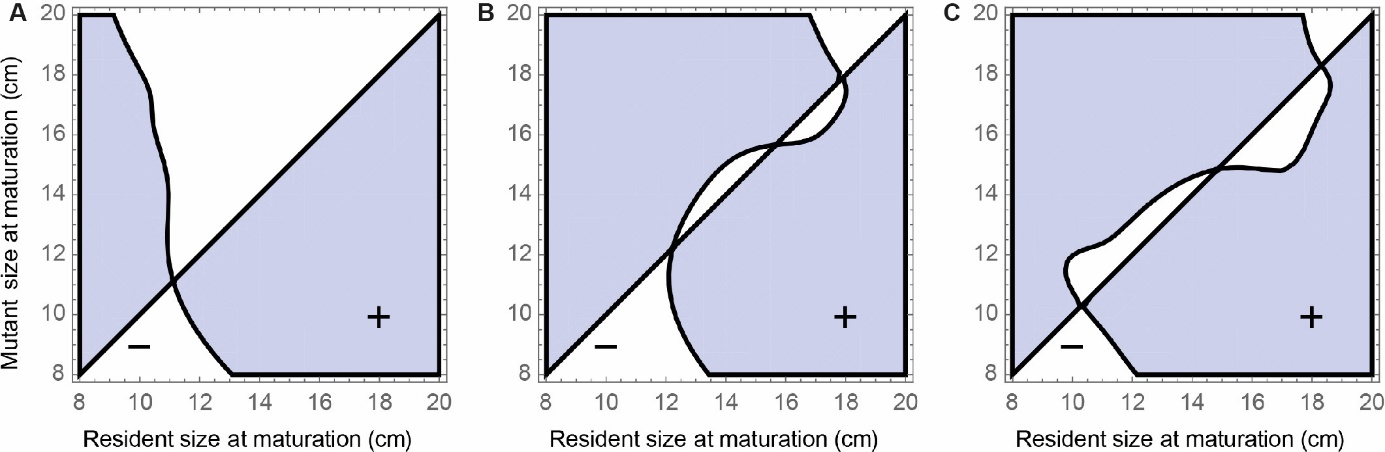


**Figure S6. Pairwise invasibility plots for three different predation intensities.** (**A**) Low predation intensity (20%) yields one evolutionarily stable maturation size. (**B**) Medium predation intensity (50%) yields two convergence stable singular strategies, where the larger one is evolutionarily stable and the smaller one is a branching point. (**C**) High predation intensity (80%) yields two evolutionarily stable maturation sizes.

**Table S1.** Genetic data and geographic location for the lakes (n=32) where micro satellites were analysed. For lakes with more than two ecotypes, we present the genetic distance between the largest and the smallest ecotype. Two of the lakes, Stor-Ringsjön and Murusjøen, have whitefish populations that are too young to be included in the interview-based analysis underlying Figure 2b, and hence, also to be used in the validation of said interview-based results that is presented in the results section.

| **Lake** | **Ecotype** | **Latitude** | **Longitude** | **Year of introduction** | **# μ-sat markers** | **# Pop Gen** | ***F*_ST_** | **HWE (*P*-Value)** |
| --- | --- | --- | --- | --- | --- | --- | --- | --- |
| Bodsjön | Giant | 62.8527 | 15.4145 |  | 19 | 22 | 0.054* | 0.97 |
|  | Intermediate |  |  |  |  | 26 |  | 0.50 |
|  | Dwarf |  |  |  |  | 26 |  | 0.82 |
| Bölessjön^†^ | Giant | 62.9263 | 14.8418 | 1825 | 9 | 29 | 0 | 0.22 |
|  | Intermediate |  |  |  |  | 20 |  | 0.94 |
|  | Dwarf |  |  |  |  | 21 |  | 0.41 |
| Femunden^‡^ | Bay spawner^‡^ | 61.9349 | 11.8633 |  | 6 | 45 | 0.120* | <0.05 |
|  | Deep spawner^‡^ |  |  |  |  | 182 |  | <0.05 |
|  | Stream spawner^‡^ |  |  |  |  | 146 |  | <0.05 |
|  | Shallow spawner^‡^ |  |  |  |  | 91 |  | <0.05 |
| Gysen^†^ | Giant | 63.6414 | 14.3934 | 1830 | 19 | 30 | 0.007* | 0.89 |
|  | Dwarf |  |  |  |  | 28 |  | 0.23 |
| Gåxsjön | Giant | 63.6729 | 15.1012 |  | 19 | 14 | 0.057* | 0.24 |
|  | Dwarf |  |  |  |  | 20 |  | 0.91 |
| Görvikssjön | Giant | 63.5989 | 15.7038 | 1845 | 9 | 27 | 0.025***** | 0.12 |
|  | Dwarf |  |  |  |  | 25 |  | 0.76 |
| Hetögeln^†^ | Giant | 64.3884 | 14.4208 | 1960 | 9 | 27 | 0 | 0.73 |
|  | Dwarf |  |  |  |  | 27 |  | 0.88 |
| Hotagen | Giant | 63.7890 | 14.6680 |  | 19 | 19 | 0.068* | 0.95 |
|  | Dwarf |  |  |  |  | 30 |  | 0.93 |
| Hökvattnet | Giant | 63.8834 | 14.8386 | 1865 | 9 | 29 | 0.064* | 0.04* |
|  | Intermediate |  |  |  |  | 27 |  | 0.14 |
|  | Dwarf |  |  |  |  | 29 |  | 0.04* |
| Idsjön | Giant | 62.8180 | 15.7121 |  | 19 | 26 | 0.055***** | 0.91 |
|  | Intermediate |  |  |  |  | 29 |  | 0.47 |
|  | Dwarf |  |  |  |  | 22 |  | 0.55 |
| Ismunden | Giant | 63.1474 | 15.1963 |  | 19 | 18 | 0.102* | 0.63 |
|  | Dwarf |  |  |  |  | 20 |  | 0.95 |
| Isteren^‡^ | ”Normal” | 61.9096 | 11.7774 |  | 6 | 30 | 0.085* | >0.05 |
|  | ”Dwarf” |  |  |  |  | 30 |  | >0.05 |
| Locknesjön | Giant | 62.9198 | 14.9395 |  | 19 | 29 | 0.022* | 0.50 |
|  | Intermediate |  |  |  |  | 15 |  | 0.93 |
|  | Dwarf |  |  |  |  | 25 |  | 0.64 |
| Långvattnet | Giant | 62.9629 | 15.1757 |  | 19 | 7 | 0.051***** | 1.00 |
|  | Dwarf |  |  |  |  | 23 |  | 0.05* |
| Murusjøen | Giant | 64.4597 | 14.1022 | 1975 | 19 | 10 | 0.011 | 0.95 |
|  | Dwarf |  |  |  |  | 15 |  | 0.54 |
| Oxvattensjön^†^ | Giant | 64.0905 | 17.8637 | 1926 | 9 | 21 | 0.001 | 0.54 |
|  | Dwarf |  |  |  |  | 20 |  | 0.44 |
| Rammsjön | Giant | 62.3789 | 14.5869 | 1930 | 9 | 30 | 0.064***** | 0.21 |
|  | Dwarf |  |  |  |  | 26 |  | 0.80 |
| Revsundssjön | Giant | 62.8140 | 15.3534 |  | 19 | 29 | 0.055* | 0.91 |
|  | Intermediate |  |  |  |  | 23 |  | 1.00 |
|  | Dwarf |  |  |  |  | 27 |  | 0.72 |
| Rissjön^†^ | Giant | 64.0289 | 17.8095 | 1926 | 9 | 28 | 0 | 0.88 |
|  | Dwarf |  |  |  |  | 25 |  | 0.44 |
| Rosången | Giant | 62.2980 | 14.9040 | 1860 | 9 | 21 | 0.055***** | 0.99 |
|  | Dwarf |  |  |  |  | 31 |  | 0.60 |
| Singsjön | Giant | 63.1206 | 15.0922 | 1784 | 9 | 27 | 0.036***** | 0.67 |
|  | Dwarf |  |  |  |  | 25 |  | 0.31 |
| Skåsjön | Giant | 62.8035 | 15.7156 |  | 19 | 7 | 0.041* | 1.00 |
|  | Intermediate |  |  |  |  | 22 |  | 0.96 |
|  | Dwarf |  |  |  |  | 29 |  | 0.65 |
| Stor-Arasjön^†^ | Giant | 64.5986 | 17.6004 | 1937 | 9 | 19 | 0.002 | 0.35 |
|  | Dwarf |  |  |  |  | 29 |  | 0.30 |
| Stor-Ringsjön^†^ | Giant | 64.2646 | 14.9974 | 1985 | 19 | 16 | 0 | 0.63 |
|  | Dwarf |  |  |  |  | 30 |  | 0.96 |
| Stor-Skirsjön^†^ | Giant | 64.0659 | 16.0843 | 1945 | 9 | 29 | 0.010 | 0.77 |
|  | Dwarf |  |  |  |  | 25 |  | 0.42 |
| Storvindeln | Giant | 65.6369 | 17.4507 |  | 19 | 23 | 0.036* | 0.17 |
|  | Interm. dens. rakered |  |  |  |  | 28 |  | 0.80 |
|  | Interm. spars. rakered |  |  |  |  | 25 |  | 0.85 |
|  | Dwarf |  |  |  |  | 21 |  | 0.38 |
| Ströms Vattudal | Giant | 63.9303 | 15.6104 | 1854 | 9 | 27 | 0.015***** | 0.06 |
|  | Dwarf |  |  |  |  | 30 |  | 0.57 |
| Sundsjön | Giant | 62.9629 | 15.1757 |  | 9 | 28 | 0.025***** | 0.90 |
|  | Dwarf |  |  |  |  | 8 |  | 0.48 |
| Sällsjön^†^ | Giant | 63.2376 | 13.6940 | 1870 | 19 | 28 | 0.024***** | 0.05 |
|  | Dwarf |  |  |  |  | 9 |  | 0.97 |
| Sörvikssjön^†^ | Giant | 63.5520 | 15.8496 | 1845 | 19 | 32 | 0.001 | 0.60 |
|  | Dwarf |  |  |  |  | 19 |  | 0.83 |
| Valsjön | Giant | 64.0198 | 14.2074 | <1900^§^ | 19 | 28 | 0.119* | 0.99 |
|  | Dwarf |  |  |  |  | 28 |  | 0.02* |
| Vallsjön | Giant | 62.9226 | 16.0656 | 1915 | 9 | 15 | 0.073***** | 0.96 |
|  | Dwarf |  |  |  |  | 28 |  | 0.83 |

* *P* < 0.05

^†^ Introduced populations of whitefish that were included in the chronosequence of lakes shown in Figs. 4 and 5 (pike present category).

^‡^ Data from Østbye et al. 2005  ([Østbye et al. 2005](#_ENREF_42))_._

§ Conflicting information about introduction date

**Table S2.** Differences between coexisting dwarf and giant whitefish ecotypes in the number of gill rakers (n=72). Two of the lakes, Stor-Ringsjön and Murusjøen, have whitefish populations that are too young to be included in the interview-based analysis underlying Figure 2b, and hence, also to be used in the validation of said interview-based results that is presented in the results section.

| **Lake** | **Year of intro-duction** | **Latitude** | **Longitude** | **Gill raker difference** | **t-value** | **p-value** | **No of**  **obs.** | **Reference** |
| --- | --- | --- | --- | --- | --- | --- | --- | --- |
| Alsensjön |  | 63.3241 | 14.1493 | 8.5 | 9.8 | <0.0001 | 67 | ([Svärdson 1977](#_ENREF_36)) |
| Ansjön |  | 62.9764 | 16.0890 | 2.3 | 18.5 | <0.0001 | 3192 | ([Svärdson 1979](#_ENREF_37)) |
| Bodsjön |  | 62.8662 | 14.9831 | 10.1 | 13.2 | <0.0001 | 69 | ([Svärdson 1979](#_ENREF_37)) |
| Bodsjön |  | 62.8527 | 15.4145 | 8.6 | 19.3 | <0.0001 | 60 | this study |
| Bomsjön | 1895 | 64.6009 | 17.1052 | 0.72 | 1.58 | 0.12 | 121 | this study |
| Bölessjön^†^ | 1825 | 62.2372 | 17.3906 | 2.33 | 4.57 | <0.0001 | 122 | this study |
| Dikasjön |  | 65.2145 | 16.0419 | 13.6 | 29.4 | <0.0001 | 158 | ([Svärdson 1998](#_ENREF_38)) |
| Femund |  | 61.9178 | 11.9445 | 16.6 | 63.1 | <0.0001 | 219 | ([Svärdson 1979](#_ENREF_37)) |
| Flåsjön |  | 64.1308 | 15.9169 | 6.6 | 10.0 | <0.0001 | 43 | this study |
| Gesunden |  | 63.1460 | 16.0698 | 4.6 | 6.8 | <0.0001 | 105 | ([Svärdson 1977](#_ENREF_36)) |
| Gysen^†^ | 1830 | 63.6414 | 14.3934 | 1.53 | 5.48 | <0.0001 | 244 | this study |
| Gåxsjön |  | 63.6729 | 15.1012 | 19.2 | 39.1 | <0.0001 | 71 | this study |
| Gärdessjön | 1908 | 63.5596 | 13.7502 | 0.30 | 0.59 | 0.24 | 65 | this study |
| Görvikssjön | 1845 | 63.5989 | 15.7038 | 7.0 | 9.5 | <0.0001 | 79 | this study |
| Hammerdalssjön |  | 63.5384 | 15.4453 | 16.2 | 28.6 | <0.0001 | 40 | ([Svärdson 1977](#_ENREF_36)) |
| Helgesjön | 1865 | 63.3400 | 13.3125 | 14.2 | 19.4 | <0.0001 | 98 | ([Svärdson 1977](#_ENREF_36)) |
| Hetögeln^†^ | 1960 | 64.3884 | 14.4208 | 0.28 | 0.64 | 0.28 | 117 | this study |
| Hornavan |  | 66.0593 | 17.8727 | 13.9 | 40.0 | <0.0001 | 425 | ([Svärdson 1979](#_ENREF_37)) |
| Hotagen |  | 63.7890 | 14.6680 | 3.9 | 8.0 | <0.0001 | 140 | ([Svärdson 1998](#_ENREF_38)) |
| Härjåsjön |  | 61.9160 | 14.1969 | 15.1 | 37.1 | <0.0001 | 90 | ([Svärdson 1979](#_ENREF_37)) |
| Hökvattnet | 1865 | 63.8834 | 14.8386 | 1.6 | 2.25 | 0.012 | 76 | this study |
| Idsjön |  | 62.8180 | 15.7121 | 5.6 | 13.4 | <0.0001 | 102 | this study |
| Ismunden |  | 63.1474 | 15.1963 | 19.6 | 39.6 | <0.0001 | 93 | this study |
| Laisan |  | 65.9918 | 17.1964 | 24.6 | 43.1 | <0.0001 | 180 | ([Svärdson 1979](#_ENREF_37)) |
| Landösjön |  | 63.5477 | 14.2856 | 4.4 | 39.9 | <0.0001 | 1862 | ([Svärdson 1977](#_ENREF_36)) |
| Locknesjön |  | 62.9198 | 14.9395 | 18.5 | 37.5 | <0.0001 | 168 | this study |
| Lossen |  | 62.4038 | 12.9249 | 3.5 | 20.2 | <0.0001 | 574 | ([Svärdson 1979](#_ENREF_37)) |
| Långvattnet |  | 65.1802 | 16.5435 | 4.4 | 6.2 | <0.0001 | 58 | this study |
| Lännässjön |  | 62.6315 | 14.1325 | 10.9 | 6.5 | <0.0001 | 17 | this study |
| Murusjøen | 1975 | 64.4597 | 14.1022 | 0.17 | 0.24 | 0.81 | 53 | this study |
| Näkten |  | 62.9118 | 14.5694 | 17.7 | 82.4 | <0.0001 | 303 | ([Svärdson 1977](#_ENREF_36)) |
| Näldsjön |  | 63.3460 | 14.2419 | 7.1 | 14.9 | <0.0001 | 276 | ([Svärdson 1977](#_ENREF_36)) |
| Ockesjön |  | 63.2782 | 13.8612 | 2.5 | 7.5 | <0.0001 | 174 | ([Svärdson 1977](#_ENREF_36)) |
| Ormsjön |  | 64.3393 | 16.1124 | 3.6 | 11.8 | <0.0001 | 348 | ([Svärdson 1979](#_ENREF_37)) |
| Orrmosjön |  | 61.8374 | 14.1175 | 16.2 | 32.5 | <0.0001 | 85 | ([Svärdson 1979](#_ENREF_37)) |
| Orten-Randsjön |  | 62.2565 | 13.8307 | 9.9 | 12.1 | <0.0001 | 55 | ([Svärdson 1979](#_ENREF_37)) |
| Ottsjön |  | 63.7073 | 14.9500 | 17.4 | 26.0 | <0.0001 | 234 | ([Svärdson 1977](#_ENREF_36)) |
| Oxvattensjön^†^ | 1926 | 64.0905 | 17.8637 | 0.25 | 0.46 | 0.64 | 119 | this study |
| Parkijaure |  | 66.7448 | 19.2048 | 22.8 | 63.3 | <0.0001 | 287 | ([Svärdson 1979](#_ENREF_37)) |
| Rammsjön | 1930 | 62.3789 | 14.5869 | 7.5 | 13.1 | <0.0001 | 68 | this study |
| Randijaure |  | 66.6579 | 19.4000 | 14.7 | 22.1 | <0.0001 | 81 | ([Petersson 1971](#_ENREF_27)) |
| Revsundssjön |  | 62.8140 | 15.3534 | 6.8 | 18.8 | <0.0001 | 82 | this study |
| Rissjön^†^ | 1926 | 64.0289 | 17.8095 | 0.81 | 1.67 | 0.10 | 76 | this study |
| Rosången | 1860 | 62.2980 | 14.9040 | 14.1 | 19.0 | <0.0001 | 77 | this study |
| Sicksjön |  | 63.0326 | 63.0302 | 11.4 | 50.5 | <0.0001 | 907 | ([Svärdson 1979](#_ENREF_37)) |
| Singsjön | 1784 | 63.1206 | 15.0922 | 10.0 | 18.7 | <0.0001 | 129 | this study |
| Skikkisjaure |  | 65.1192 | 16.4243 | 17.6 | 36.9 | <0.0001 | 113 | ([Svärdson 1957](#_ENREF_35)) |
| Skåsjön |  | 62.8035 | 15.7156 | 14.1 | 15.3 | <0.0001 | 81 | this study |
| Stor-Arasjön^†^ | 1937 | 64.5986 | 17.6004 | 0.68 | 1.12 | 0.27 | 63 | this study |
| Stora Skeppsträsk |  | 65.1857 | 18.9198 | 10.3 | 24.1 | <0.0001 | 443 | ([Svärdson 1957](#_ENREF_35)) |
| Storavan |  | 65.6740 | 18.1433 | 12.5 | 51.8 | <0.0001 | 259 | ([Svärdson 1979](#_ENREF_37)) |
| Stor-Juktan |  | 65.3210 | 17.3313 | 8.5 | 19.3 | <0.0001 | 77 | ([Hammar 1988](#_ENREF_17)) |
| Storsjön |  | 63.2983 | 14.4601 | 15.7 | 46.5 | <0.0001 | 169 | ([Svärdson 1953](#_ENREF_34)) |
| Stor-Skirsjön^†^ | 1945 | 64.0659 | 16.0844 | 0.25 | 0.66 | 0.51 | 126 | this study |
| Stor-Ringsjön^†^ | 1985 | 64.2646 | 14.9974 | 0.61 | 1.13 | 0.27 | 54 | this study |
| Storuman |  | 65.0946 | 17.1007 | 21.2 | 114.2 | <0.0001 | 958 | ([Svärdson 1979](#_ENREF_37)) |
| Storvindeln |  | 65.6369 | 17.4507 | 16.3 | 38.9 | <0.0001 | 119 | this study |
| Ströms Vattudal | 1854 | 63.9303 | 15.6104 | 2.4 | 4.2 | 0.0009 | 113 | this study |
| Sundsjön |  | 62.9629 | 15.1757 | 15.6 | 17.1 | <0.0001 | 41 | this study |
| Sällsjön^†^ | 1870 | 63.2376 | 13.6939 | 2.71 | 5.95 | <0.0001 | 142 | this study |
| Sörvikssjön^†^ | 1845 | 63.5520 | 15.8496 | 1.47 | 3.23 | 0.0017 | 108 | this study |
| Tåsjön |  | 64.1607 | 16.0072 | 9.1 | 7.1 | <0.0001 | 30 | this study |
| Uddjaure |  | 65.8464 | 18.0429 | 16.8 | 12.4 | <0.0001 | 15 | this study |
| Vallsjön | 1915 | 62.9226 | 16.0656 | 10.9 | 15.0 | <0.0001 | 51 | this study |
| Valsjön | <1900^§^ | 64.0198 | 14.2074 | 0.76 | 2.34 | 0.021 | 98 | this study |
| Venjanssjön |  | 60.8357 | 14.1193 | 3.7 | 8.4 | <0.0001 | 106 | ([Svärdson 1979](#_ENREF_37)) |
| Vikarsjön |  | 62.3750 | 13.7589 | 7.9 | 15.9 | <0.0001 | 92 | ([Svärdson 1979](#_ENREF_37)) |
| Vojmsjön |  | 64.8618 | 16.7438 | 16.3 | 130.5 | <0.0001 | 1861 | ([Svärdson 1979](#_ENREF_37)) |
| Volgsjön |  | 64.5501 | 16.7145 | 13.9 | 28.8 | <0.0001 | 431 | ([Svärdson 1957](#_ENREF_35)) |
| Östra Vattnan |  | 62.3339 | 12.6695 | 10.1 | 17.0 | <0.0001 | 28 | ([Svärdson 1979](#_ENREF_37)) |
| Över-Särvsjön |  | 62.6319 | 13.1551 | 15.6 | 36.9 | <0.0001 | 177 | ([Svärdson 1979](#_ENREF_37)) |
| Övsjön |  | 63.0302 | 15.9887 | 1.2 | 3.2 | 0.0016 | 174 | ([Svärdson 1979](#_ENREF_37)) |

^§^ Conflicting information about introduction date

^†^ Introduced populations of whitefish that were included in the chronosequence of lakes shown in Figs. 4 and 5 (pike present category).

**Table S3.** Selection of lakes for the analyses in the paper. As a general rule, each analysis included all lakes that were relevant for the stated hypothesis and for which we had relevant data. Exceptions to this rule were made in Figs 2a, 2b and Figs S1 and S2, where lakes with recently introduced (after 1960) whitefish were excluded.

| **Figure/analysis** | **Total n** | **Data selection** |
| --- | --- | --- |
| 2a | 320 | Excluded: lakes that had whitefish introduced after 1960 (n=2), belonged to category three (i.e. had uncertain status regarding mono-/polymorphism, n=6), lacked pike and had polymorphic whitefish (n=5), or had missing data (n=25). |
| 2b | 350 | Excluded: lakes that belonged to category three (n=6), or had whitefish introduced after 1960 (n=2). |
| 3 | 38 | Included: all lakes with dated whitefish introductions where we had standardized sample fishing data |
| 4 | 19 | Included: (1) all lakes that met the following three criteria: a) there were known, dated whitefish introductions, b) we had microsatellite data suggesting no secondary introductions, and c) we had performed standardized sample fishing and cluster analysis on the resulting body size- and gill raker data gave significant clusters (n=10), and (2) all lakes with native whitefish where we had performed standardized sample fishing and counted gill rakers (n=9) |
| 5 | 23 | Included: all lakes (n=23) where the standardized gillnet sampling setup had been extended with two extra floating gillnets of 33 and 45 mm mesh size (allowing the average size of sexually mature whitefish to be compared between habitats using data originating from the same mesh sizes). This selection includes the same 10 young pike presence lakes that were included in Fig. 4, 10 pike-less control lakes and 3 lakes with native polymorphic whitefish. |
| 6 | 72 | Included: all populations (n=72) for which we could get information about spawning habitat and average body size and gill raker number, and that came from lakes with an area ≥100 ha and with a maximum depth ≥ 15 m. |
| Fig S1 | 350 | Excluded: lakes that belonged to category three (n=6), or had whitefish introduced after 1960 (n=2). |
| Fig S2 | 191 | Included: All lakes in category four (monomorphic) except lakes that had whitefish introduced after 1960 (n=2) or had missing data (n=6) |

| **Predictor** | **Variable**  **importance** |
| --- | --- |
| Pike | 24 |
| Lake area | 24 |
| Lake depth | 12 |
| Perch | 11 |
| Arctic char | 10 |
| Species richness | 9 |
| Degree days | 6 |
| Elevation | 5 |
| Roach | <1 |
| Brown trout | <1 |
| Lake trout | <1 |
| Grayling | <1 |
| Burbot | <1 |

**Table S4.** Variable importance for the predictors included in the classification analyses.

**Table S5.** Description of the model parameters. Superscript numbers within parentheses refers to Supplementary References.

|  | | | | |  | |  |  |
| --- | --- | --- | --- | --- | --- | --- | --- | --- |
| **Constant** | **Value** | | **Description (reference)** | | | **Unit** | |  |
| Prey physical parameters | | | | | | |  |  |
| *S_0_* | 1.4 | | Size at birth ([Andersson 2005](#_ENREF_1)) | | | cm | | |
| *λ_1_* | 5.03 | | Length to weight relation ([Andersson 2005](#_ENREF_1)) | | | $\mathrm{cm}g^{-\lambda_{2}}$ | | |
| *λ_2_* | 0.32 | | Length weight exponent ([Andersson 2005](#_ENREF_1)) | | | − | | |
| *ξ_1_* | 5.33 | | Handling time constant ([Andersson 2005](#_ENREF_1)) | | | $\mathrm{day}g^{-(1+\xi_{2})}$ | | |
| *ξ_2_* | -0.66 | | Handling time exponent ([Andersson 2005](#_ENREF_1)) | | | − | | |
| *β_1_* | 0.033 | | Metabolic rate constant ([Persson et al. 1998](#_ENREF_26)) | | | $\mathrm{day}^{-1} g^{-(1+\beta_{2})}$ | | |
| *β_2_* | 0.7 | | Metabolic rate exponent ([De Roos et al. 1992](#_ENREF_7)) | | | − | | |
| *ε* | 0.61 | | Conversion coefficient ([Andersson 2005](#_ENREF_1)) | | | − | | |
| *η* | 1 | | Energy cost for producing one egg | | | g | | |
| *q* | 0.02 | | Probability of surviving the larval stage | | | − | | |
| *k* | 5 | | Energy allocation slope constant | | | cm^-1^ | | |
| *𝜅_j_* | 1 | | Juvenile energy allocation for growth | | | − | | |
| *𝜅_a_* | 0.04 | | Adult energy allocation for growth | | | − | | |
| *θ* | 5 | | Strength of habitat selection | | | − | | |
|  | |  | |  |  | |  |  |
| Pelagic habitat (Habitat 1) | | | | | | |  |  |
| r_1_ | 0.1 | | Growth rate of the zooplankton resource *R_1_*  ([Andersson 2005](#_ENREF_1)) | | | day^-1^ | |  |
| *K_1_* | 1.0 | | Carrying capacity of resource *R_1_* ([Evans et al. 1996](#_ENREF_9)) | | | g m^-3^ | |  |
| *V_1_* | 10^6^ | | Volume of the pelagic habitat | | | m^3^ | |  |
| $\hat{a}_{1}$ | 14.0 | | Maximum attack rate on resource *R_1_*  ([Andersson 2005](#_ENREF_1)) | | | m^3^ d^-1^ | |  |
| *α_1_* | 0.65 | | Attack rate exponent for *R_1_* | | | − | |  |
| *W_0_* | 15 | | Weight at optimal attack rate on resource *R_1_* | | | g | |  |
| *m_1_* | 0.0025 | | Background mortality rate in the pelagic habitat | | | day^-1^ | |  |

|  | | | |  |
| --- | --- | --- | --- | --- |
| Littoral habitat (Habitat 2) | | | |  |
| *r_2_* | 0.1 | Growth rate of macroinvertebrate resource *R_2_* ([Andersson 2005](#_ENREF_1)) | day^-1^ | |
| *K_2_* | 28.0 | Carrying capacity of resource *R_2_* ([Rasmussen 1988](#_ENREF_30)) | g m^-2^ | |
| *V_2_* | 50000 | Area of the littoral habitat ([Persson et al. 1998](#_ENREF_26)) | m^2^ | |
| $\hat{a}_{2}$ | 0.6 | Attack rate constant for resource *R_2_* ([Andersson 2005](#_ENREF_1)) | m^2^ day^-1^ | |
| *α_2_* | 0.3 | Attack rate exponent *R_2_* ([Andersson 2005](#_ENREF_1)) | − | |
| *m_2_* | 0.0025 | Background mortality rate in the littoral habitat | day^-1^ | |
| *k_p_* | 5 | Predation slope constant at maximum gape size | cm^-1^ | |
| *γ_p_* | 1 | Predation mortality exponent | − | |
| *I_p_* | Varied | Maximum predator gape size | cm | |
| *p* | Varied | Predator attack rate constant | day^-1^ | |
|  | | | |  |

**Table S6.** Description of the model variables and functions.

| **Variable** | **Description** | **Unit** |
| --- | --- | --- |
| *S* | Size | cm |
| *s_m_* | Maturation size | cm |
| *n(s,t)* | Population size distribution at time $t$ | cm^-1^ |
| *R_1_* | Density of the pelagic resource | g m^-3^ |
| *R_2_* | Density of the littoral resource | g m^-2^ |

| **Function** |  |  |
| --- | --- | --- |
| $l(w)=\lambda_{1}w^{\lambda_{2}}$ (below *w=*$l$*^-1^*(*s*)) | Length of an individual with weight $W$ gram | cm |
| $\kappa\left( s \right)=\kappa_{j}-\frac{(\kappa_{j}-\kappa_{a})}{1+exp[-k\left( s-s_{m} \right)]}$ | Fraction of energy channelled to growth | − |
| $a_{1}\left( s \right)=\hat{a}_{1}\left( \frac{W}{W_{0}}\exp\left[ 1-\frac{W}{W_{0}} \right] \right)^{\alpha_{1}}$ | Attack rate on resource *R_1_* | m^3^ day^-1^ |
| $a_{2}(s)=\hat{a}_{2}w^{\alpha_{2}}$ | Attack rate on resource *R_2_* | m^2^ day^-1^ |
| $H(s)=\xi_{1}w^{\xi_{2}}$ | Handling time at size $s$ | day g^-1^ |
| $p(s)=\left( \frac{p}{s} \right)^{\gamma_{p}}\left( s_{0}-\frac{s_{0}}{1+\exp\left[ -k_{p}(s-l_{p}) \right]} \right)$ | Predation mortality rate at size $s$ | day^-1^ |
| $\mu_{1}\left( s \right)=m_{1}$ | Mortality in habitat 1 | day^-1^ |
| $\mu_{2}\left( s \right)=m_{2}+p(s)$ | Mortality in habitat 2 | day^-1^ |
| $T_{i}\left( s,\mathbf{R} \right)\boldsymbol{=}\frac{{{(a}_{i}(s)R_{i}/\mu_{i}(s))}^{\theta}}{{{(a}_{1}(s)R_{1}/\mu_{1}(s))}^{\theta}+{{(a}_{2}(s)R_{2}/\mu_{2}(s))}^{\theta}}$ | Fraction of time spent foraging in habitat $i$ | − |
| $I_{i}\left( s,\mathbf{R} \right)\boldsymbol{=}\frac{a_{i}(s)R_{i}}{1+H(s)a_{i}(s)R_{i}}$ | Food intake rate from resource *R_i_* when feeding only on *R_i_* | g day^-1^ |
| $f_{i}\left( s,\mathbf{R} \right)\boldsymbol{=}T_{i}\left( s,\mathbf{R} \right)I_{i}\left( s,\mathbf{R} \right)$ | Food intake rate from resource *R_i_* | g day^-1^ |
| $f\left( s,\mathbf{R} \right)=f_{1}\left( s,\mathbf{R} \right)\boldsymbol{+}f_{2}\left( s,\mathbf{R} \right)$ | Total food intake rate | g day^-1^ |
| $g\left( s,\mathbf{R} \right)=\kappa\left( s \right)\frac{\lambda_{2}s}{\left( s/{\lambda_{1}} \right)^{1/{\lambda_{2}}}}(\varepsilon f\left( s,\mathbf{R} \right)-\beta_{1}w^{\beta_{2}})$ | Growth rate | cm day^-1^ |
| $b\left( s,\mathbf{R} \right)=\frac{q(1-\kappa\left( s \right))}{\eta}(\varepsilon f\left( s,\mathbf{R} \right)-\beta_{1}w^{\beta_{2}}$) | Birth rate | day^-1^ |
| $d\left( s,\mathbf{R} \right)=T_{1}\left( s,\mathbf{R} \right)\mu_{1}(s)\boldsymbol{+}T_{2}\left( s,\mathbf{R} \right)\mu_{2}(s)$ | Death rate | day^-1^ |

**Table S7.** Robustness of model results. A range of parameters was tested to determine the robustness of the conclusion that predation induces evolutionary divergence. The parameters are presented in descending order of sensitivity. Each parameter was tested for 10 or 11 equally spaced values for predation intensities 15%, 30%, 45%, 60%, and 75%, with gape size set to 18 cm.

| **Parameter** | **Value** | **Tested range** | **Range producing divergence** | **Proportion of runs giving divergence** |
| --- | --- | --- | --- | --- |
| $\eta$ | 1 | [0.2,2] | [0.4,1.4] | 56 % |
| $w_{0}$ | 15 | [5,30] | [7.5,22.5] | 60 % |
| $q$ | 0.02 | [0.005,0.05] | [0.015,0.05] | 78 % |
| $\theta$ | 5 | [0,9] | [2,9] | 78 % |
| $m_{1}$ | 0.0025 | [0.0005,0.005] | [0.0005,0.004] | 78 % |
| $m_{2}$ | 0.0025 | [0.0005,0.005] | [0.0005,0.004] | 78 % |
| $\gamma_{p}$ | 1 | [0.5,1.5] | [0.6,1.5] | 90 % |
| $\kappa_{a}$ | 0.04 | [0.01,0.1] | [0.01,0.1] | 100 % |
| $k$ | 5 | [1,10] | [1,10] | 100 % |
| $k_{p}$ | 5 | [1,10] | [1,10] | 100 % |

**Supplemental References**

Andersson, J. 2005. The development of resource polymorphism - effects of diet, predation risk and population dynamical feedbacks. Ph. D. thesis. Umeå University, Umeå.

Brännstrom, Å., Carlsson, L., & Simpson, D. 2013. On the convergence of the escalator boxcar train Siam J. Num. Anal. 51:3213-3231.

Byström, P., & Andersson, J. 2005. Size-dependent foraging capacities and intercohort competition in an ontogenetic omnivore (Arctic char). Oikos 110:523-536.

Claessen, D., & Dieckmann, U. 2002. Ontogenetic niche shifts and evolutionary branching in size-structured populations. Evol. Ecol. Res. 4:189-217.

Clarke, A., & Johnston, N. M. 1999. Scaling of metabolic rate with body mass and temperature in teleost fish. J. Anim. Ecol. 68:893-905.

de Roos, A. M. 1997. A gentle introduction to physiologically structured population models. Population and Community Biology Series 18:119-204.

De Roos, A. M., Diekmann, O., & Metz, J. A. J. 1992. Studying the dynamics of structured population models: a versatile technique and its application to *Daphnia*. Am. Nat. 139:123-147.

Dieckmann, U., & Law, R. 1996. The dynamical theory of coevolution: A derivation from stochastic ecological processes. J. Math. Biol. 34:579-612.

Evans, M. S., Arts, M. T., & Robarts, R. D. 1996. Algal productivity, algal biomass, and zooplankton biomass in a phosphorus-rich, saline lake: Deviations from regression model predictions. Can. J. Fish. Aquat. Sci. 53:1048-1060.

Excoffier, L., Laval, G., & Schneider, S. 2005. Arlequin (version 3.0): An integrated software package for population genetics data analysis. Evol. Bioinform. 1:47-50.

Falush, D., Stephens, M., & Pritchard, J. K. 2003. Inference of population structure using multilocus genotype data: Linked loci and correlated allele frequencies. Genetics 164:1567-1587.

Felsenstein, J. 1989. PHYLIP - Phylogeny Inference Package (Version 3.2). Cladistic 5:164-166.

Filipsson, O., & Svärdson, G. 1976. Principles for the management of char populations. Inf. Inst. Freshw. Res. Drottningholm 2:1-79.

Francis, R. M. 2017. POPHELPER: an R package and web app to analyse and visualize population structure. Mol. Ecol. Resour. 17:27-32.

Geritz, S. A. H., Kisdi, E., Meszena, G., & Metz, J. A. J. 1998. Evolutionarily singular strategies and the adaptive growth and branching of the evolutionary tree. Evol. Ecol. 12:35-57.

Gilliam, J. F., & Fraser, D. F. 1987. Habitat selection under predation hazard: test of a model with foraging minnows. Ecology 68:1856-1862.

Hammar, J. 1988. Planktivorous whitefish and introduced *Mysis relicta*: Ultimate competitors in the pelagic community. Finnish Fisheries Research 9:497-521.

Kahilainen, K., Lehtonen, H., & Kononen, K. 2003. Consequence of habitat segregation to growth rate of two sparsely rakered whitefish (*Coregonus lavaretus* (L.)) forms in a subarctic lake. Ecol. Freshw. Fish 12:275-285.

Kopelman, N. M., Mayzel, J., Jakobsson, M., Rosenberg, N. A., & Mayrose, I. 2015. Clumpak: a program for identifying clustering modes and packaging population structure inferences across K. Mol. Ecol. Resour. 15:1179-1191.

L'Abee-Lund, J. H., Langeland, A., Jonsson, B., & Ugedal, O. 1993. Spatial segregation by age and size in Arctic charr: A trade-off between feeding possibility and risk of predation. J. Anim. Ecol. 62:160-168.

Metz, J. A. J., & Diekmann, O. (1986). The dynamics of physiologically structured populations. Springer, Berlin.

Metz, J. A. J., Nisbet, R. M., & Geritz, S. A. H. 1992. How should we define ’fitness’ for general ecological scenarios? Trends Ecol. Evol. 7:198-202.

Nilsson, N. A., & Filipsson, O. 1971. Characteristics of two discrete populations of Arctic char (*Salvelinus alpinus* L. ) in a north Swedish lake. Rep. Inst. Fresw. Res, Drottningholm 51:90-108.

Nosil, P., Harmon, L. J., & Seehausen, O. 2009. Ecological explanations for (incomplete) speciation. Trends Ecol. Evol. 24:145-156.

Olofsson, O. 1934. Några inplanteringar av Lomsjö-sik. Svensk Fiskeritidskrift 43:16-18.

Persson, L., Leonardsson, K., De Roos, A. M., Gyllenberg, M., & Christensen, B. 1998. Ontogenetic scaling of foraging rates and the dynamics of a size-structured consumer-resource model. Theor. Pop. Biol. 54:270-293.

Petersson, Å. 1971. The effect of lake regulation on populations of Cestodan parasites of Swedish whitefish, *Coregonus*. Oikos 22:74-83.

Pew, J., Muir, P. H., Wang, J., & Frasier, T. R. 2015. Related: an R package for analysing pairwise relatedness from codominant molecular markers. Mol. Ecol. Resour. 15:557-561.

Præbel, K., Westgaard, J. I., Amundsen, P. A., Siwertsson, A., Knudsen, R., Kahilainen, K. K., & Fevolden, S. E. 2013. A diagnostic tool for efficient analysis of the population structure, hybridization and conservation status of European whitefish (*Coregonus lavaretus* (L.)) and vendace (*C. albula* (L.)). Pp. 247-255 *in* J. Wanzenbock and I. J. Winfield, eds. Biology and Management of Coregonid Fishes - 2011.

Rasmussen, J. B. 1988. Littoral zoobenthic biomass in lakes, and its relationship to physical, chemical, and trophic factors. Can. J. Fish. Aquat. Sci. 45:1436-1447.

Rice, W. R. 1989. Analyzing tables of statistical tests. Evolution 43:223-225.

Rousset, F. 2008. GENEPOP ' 007: a complete re-implementation of the GENEPOP software for Windows and Linux. Mol. Ecol. Resour. 8:103-106.

Skurdal, J., Bleken, E., & Stenseth, N. C. 1985. Cannibalism in whitefish (*Coregonus lavaretus*). Oecologia 67:566-571.

Svärdson, G. 1953. The Coregonid problem V. Svmpatric whitefish species of the lakes Idsjön, Storsjön and Hornavan. Rep. Inst. Fresw. Res, Drottningholm 34:141-166.

Svärdson, G. 1957. The coregonid problem. VI. The palearctic species and their intergrades. Rep. Inst. Fresw. Res, Drottningholm 38:267-356.

Svärdson, G. 1977. Sällsjösiken och de fem sikarna I Indalsälven. Information från Sötvattenslaboratoriet, Drottningholm 14:1-41.

Svärdson, G. 1979. Speciation in Scandinavian *Coregonus*. Rep. Inst. Fresw. Res, Drottningholm 57:1-95.

Svärdson, G. 1998. Postglacial dispersal and reticulate evolution of Nordic coregonids. Nordic J. Freshw. Res. 74:3-32.

Tonn, W. M., Paszkowski, C. A., & Holopainen, I. J. 1992. Piscivory and recruitment: mechanisms structuring prey populations in small lakes. Ecology 73:951-958.

Wang, J. 2007. Triadic IBD coefficients and applications to estimating pairwise relatedness. Genetical Research 89:135-153.

Werner, E. E., Gilliam, J. F., Hall, D. J., & Mittelbach, G. G. 1983. An experimental test of the effects of predation risk on habitat use in fish. Ecology 64:1540-1548.

Østbye, K., Næsje, T. F., Bernatchez, L., Sandlund, O. T., & Hindar, K. 2005. Morphological divergence and origin of sympatric populations of European whitefish (*Coregonus lavaretus* L.) in Lake Femund, Norway. J. Evol. Biol. 18:683-702.
